# Parasite reliance on its host gut microbiota for nutrition and survival

**DOI:** 10.1038/s41396-022-01301-z

**Published:** 2022-08-08

**Authors:** Sicong Zhou, Yueqi Lu, Jiani Chen, Zhongqiu Pan, Lan Pang, Ying Wang, Qichao Zhang, Michael R. Strand, Xue-Xin Chen, Jianhua Huang

**Affiliations:** 1 Institute of Insect Sciences, College of Agriculture and Biotechnology, Zhejiang University, Hangzhou, 310058 China; 2 Ministry of Agriculture Key Lab of Molecular Biology of Crop Pathogens and Insect Pests, Zhejiang University, Hangzhou, 310058 China; 3 Key Laboratory of Biology of Crop Pathogens and Insects of Zhejiang Province, Zhejiang University, Hangzhou, 310058 China; 4 Department of Entomology, University of Georgia, Athens, GA 30602 USA; 5 State Key Lab of Rice Biology, Zhejiang University, Hangzhou, 310058 China

**Keywords:** Animal physiology, Microbial ecology

## Abstract

Studying the microbial symbionts of eukaryotic hosts has revealed a range of interactions that benefit host biology. Most eukaryotes are also infected by parasites that adversely affect host biology for their own benefit. However, it is largely unclear whether the ability of parasites to develop in hosts also depends on host-associated symbionts, e.g., the gut microbiota. Here, we studied the parasitic wasp *Leptopilina boulardi* (Lb) and its host *Drosophila melanogaster*. Results showed that Lb successfully develops in conventional hosts (CN) with a gut microbiota but fails to develop in axenic hosts (AX) without a gut microbiota. We determined that developing Lb larvae consume fat body cells that store lipids. We also determined that much larger amounts of lipid accumulate in fat body cells of parasitized CN hosts than parasitized AX hosts. CN hosts parasitized by Lb exhibited large increases in the abundance of the bacterium *Acetobacter pomorum* in the gut, but did not affect the abundance of *Lactobacillus fructivorans* which is another common member of the host gut microbiota. However, AX hosts inoculated with *A. pomorum* and/or *L. fructivorans* did not rescue development of Lb. In contrast, AX larvae inoculated with *A. pomorum* plus other identified gut community members including a *Bacillus* sp. substantially rescued Lb development. Rescue was further associated with increased lipid accumulation in host fat body cells. Insulin-like peptides increased in brain neurosecretory cells of parasitized CN larvae. Lipid accumulation in the fat body of CN hosts was further associated with reduced Bmm lipase activity mediated by insulin/insulin-like growth factor signaling (IIS). Altogether, our results identify a previously unknown role for the gut microbiota in defining host permissiveness for a parasite. Our findings also identify a new paradigm for parasite manipulation of host metabolism that depends on insulin signaling and the gut microbiota.

## Introduction

Infectious organisms, including protozoan parasites and pathogenic bacteria, have long been recognized as major human health challenges [1, 2]. It is now appreciated that intimate interactions between hosts and other species are extremely widespread, and sometimes benefit hosts as exemplified by commensal gut microbes [3–5]. During the last two decades, the study of the gut microbiota has become one of the most intriguing areas of biological research. Reasons include that gut microbes are widely reported to play crucial roles in host immunity, metabolism and development [6–8]. Recently, the gut microbiota has also been found to provide a protective barrier to suppress parasitic infections through either direct competition or indirectly stimulating host immune pathways [9–12]. As such, understanding the interactions between the host microbiota and parasites could provide significant insights into interactions with hosts and potential therapeutic approaches.

Parasites are well known to use hosts as their primary source of nutrition, often exchanging their ability to synthesize nutrients *de novo* for efficient salvaging mechanisms [13–15]. Nutrients act not only as primary sources of energy but also as regulators of gene expression, metabolism and growth, through various signaling networks that enable parasites to survive [1, 16]. Evolved nutritional strategies can also modify host metabolism to suit specific nutritional requirements of the parasite [17–20]. Similar to some protozoan parasites, the offspring of parasitic wasps (also known as parasitoids) acquire their nutrients directly from the host throughout their development [21, 22]. Parasitoids also often manipulate host metabolism to meet dietary needs and promote growth and survival [23, 24]. Although alteration of host energy homeostasis by parasites is a widespread phenomenon, underlying mechanisms remain largely unknown.

Lipid stores crucially affect both host and parasite physiology [20, 25, 26]. Neutral lipids or triacylglycerols (TAGs) are primarily stored in cytosolic lipid droplets (LDs) within adipose tissue in mammals or the fat body in insects [27, 28]. Research over the past decade has established LDs as dynamic organelles and as central, active players in lipid homeostasis [29, 30]. The mechanisms underlying LD size and content include regulation of the balance between lipogenesis and lipolysis and are broadly conserved from insects to mammals [31–33]. In *Drosophila*, lipid homeostasis is substantially influenced by a group of neurosecretory cells in the brain that produce insulin-like peptides (Dilps) [34, 35]. These peptides activate the *Drosophila* insulin receptor to alter the mobilization of TAG stores in a tissue-specific manner. Upregulation of *Drosophila* insulin signaling specifically in the fat body increases TAG stores [35]. Conversely, a reduction in insulin signaling promotes nuclear translocation and transcriptional activity of Forkhead Box O (FOXO), which in turn leads to lipolysis by upregulation of Brummer (Bmm) lipase, the fly homolog of adipose triglyceride lipase (ATGL) in mammals [33].

*Leptopilina boulardi* (Lb) is a larval-pupal parasitoid. Adult females lay a single egg per oviposition inside a *Drosophila* *melanogaster* 2nd instar larva. After hatching, the Lb larva develops through five instars before pupating and emerging as an adult 19–21 days later [36, 37]. Lb provides an excellent research system for studying host-parasite interactions generally, and is also directly relevant to agricultural pest control since many parasitoids are used as biological control agents. In this study, we report that Lb develop into adults in conventional (CN) *D. melanogaster* that have a gut microbiota but do not develop in axenic (AX) hosts with no gut microbiota. Parasitism stimulated increased lipid accumulation in the fat body of CN but not AX hosts through increased insulin signaling. The mixed community of microbes in CN hosts substantially rescued Lb development when transferred to AX hosts. Screening assays indicated that transfer of two gut community members in the genus *Acetobacter* and *Bacillus* to AX *D. melanogaster* resulted in gnotobiotic (GN) hosts that also substantially rescued Lb development.

## Materials and methods

### *Drosophila* and wasp strains

*Drosophila* stocks were maintained on standard cornmeal/molasses/agar medium at room temperature. *Leptopilina boulardi* wasps were bred on a *D. melanogaster W*^*1118*^ stock, and adult wasps were maintained at room temperature in vials with apple juice agar [36]. The following *D. melanogaster* stains were used: *UAS-InR RNAi* (THU5741), *UAS-AkhR RNAi* (TH02253.N) and *UAS-Pten RNAi* (THU0582) lines were obtained from the TsingHua Fly Center (TH). *W*^*1118*^ (BL5905), *Tub-GFP-PH* (BL8164) and *Dilp2-Gal4* (BL37516) lines were obtained from the Bloomington Drosophila Stock Center (BL). *Ppl*-*Gal4* and *UAS-EGFP* lines were provided by S. Li (South China Normal University, Guangzhou, China). *UAS-Bmm RNAi*, *UAS-Lipin RNAi*, *UAS-DGAT RNAi*, *UAS-Lsd1RNAi*, and *UAS-Hsl RNAi* lines were provided by X. Huang (Institute of Genetics and Developmental Biology, Chinese Academy of Sciences, Beijing, China) [30, 38]. Each of the RNAi lines used in the study were validated by comparing relative expression levels of the target gene in fat cells to a positive control by quantitative reverse transcription PCR as described below (Supplementary Fig. 1).

### Generation of axenic host animals (AX)

Axenic larvae (AX) were generated as previously described with minor modifications [39]. *D. melanogaster* embryos were incubated for 2 min in 2.5% sodium hypochlorite, and were subsequently washed twice in 70% ethanol, followed by three washes with sterile water. The embryos were then transferred onto sterile food and routinely checked for bacterial contamination by culturing the homogenates on LB and MRS agar plates. To compensate for the developmental delay observed in axenic larvae, the yeast content was increased to 41.7 g per liter in axenic food comparing to the regular fly food recipe. Similar to the previous report [40], there was no difference in size and development time between AX and conventional (CN) *Drosophila* hosts when they were fed on the yeast-rich fly food used in this study.

### Lb parasitism and developmental stages of offspring

Mated 3-day-old Lb female wasps were allowed to parasitize CN and AX 2nd instar *D. melanogaster W*^*1118*^ at a wasp/host ratio of 1:10 for 3 h, respectively. Ten CN or AX host larvae were randomly selected and dissected under a microscope and the portion of parasitized hosts was calculated as the percentage that contained a wasp egg(s) relative to the total number of hosts that were dissected. Eight replicates were performed for the each group. The remaining parasitized hosts were collected, and the different developing stages of hosts, their fat bodies and wasp offspring were photographed using an Olympus SZX16 microscope. The developmental stages of wasps in CN or AX host larvae (Supplementary Table 1) were identified using previously described morphological characters [41].

### LD staining and size measurement

At least 20 *D. melanogaster* larvae were dissected in PBS to collect fat body and Lb larvae that were fixed in 4% paraformaldehyde in PBS for 30 min. After rinsing in PBS, samples were incubated for 30 min in a 1:1000 dilution 1 mg/ml BODIPY 493/503 (Invitrogen) in PBS, and then rinsed twice with PBS containing 0.05% Tween 20, 0.1% Triton (PBST). Stained samples were mounted in ProLong Gold Antifade Mountant with DAPI (Invitrogen). Fluorescence images were captured on a Zeiss LSM 800 confocal microscope. LD size was measured as described [30]. Briefly, the diameters of at least 150 LDs (the five largest LDs within one cell) were measured by Zeiss Zen Software version 2.3.

### Lipid measurement

TAG measurements were performed as described [42]. Fat bodies of at least six *D. melanogaster* larvae were collected and homogenized in PBST. Samples were centrifuged at 12,000 rpm for 10 min and the supernatants were heated at 70 °C for 15 min. Lipid levels were measured by using TAG Determination Kit (Sigma), and were normalized to protein amounts in each homogenate that were determined by Bradford assay (Invitrogen).

### Feeding assays

Synchronized 3rd instar *Drosophila* larvae (parasitized and nonparasitized) were cultured on fresh agar medium followed by fasting for 2 h. Thirty larvae of each group were allowed to feed on yeast diet for 20 min that contained 0.01 g bromophenol blue (FD&C Blue NO.1, TCL)/ml of water. Larvae containing varying amounts of blue color in their abdomens were categorized by visual inspection as fully-fed, partially-fed or non-fed.

### Immunohistochemistry

Fat bodies or brains from *D. melanogaster* 3rd instars were dissected in PBS and fixed in 4% paraformaldehyde in PBS for 30 min. After rinsing in PBST, tissues were blocked with 5% bovine serum albumin (BSA) in PBST, and incubated overnight at 4 °C with rabbit anti-FOXO (1:500; Cosmo Bio, Japan), rabbit anti-Dilp2 (1:500; provided by C. Tong, Zhejiang University, China), rabbit anti-NPF (1:200; provided by Z. Zhao, China Agriculture University, China), or rabbit anti-sNPF (1:200, generated against the peptides DPSLPQMRRTAYDDLLEREL by ABclonal, China). Fat bodies or brains were then washed three times in PBST and incubated with Alexa Fluor 488 or Alexa Fluor 594 secondary antibodies (1:1000; Molecular Probes) for 2 h at room temperature. Samples were washed twice in PBST, once in PBS and mounted in ProLong Gold Antifade Mountant with DAPI (Invitrogen). Fluorescence images were captured on a Zeiss LSM 800 confocal microscope, and were processed using ImageJ (National Institutes of Health) and Photoshop (Adobe).

### Lipase activity assay

Fat bodies of 20 *D. melanogaster* 3rd instars were dissected and homogenized in 100 μl lipase assay buffer. Lipase activity was measured by using Lipase Activity Kit (Sigma) according to manufacturer protocol, and was normalized to protein amounts in each homogenate by using the BCA Protein Assay Kit (Invitrogen). Experiments were repeated at least three times.

### Reverse Transcription Quantitative PCR (RT-qPCR)

Total RNA was isolated from wasp larvae as well as host brains, fat bodies, guts and the remainder of the body (carcass) using the RNeasy Mini Kit (Qiagen) according to the manufacturer’s protocol. RT-qPCR assays were performed using the AriaMx real-time PCR system (Agilent Technologies) with the ChamQ SYBR qPCR Master Mix Kit (Vazyme, Nanjing, China). Primers for amplifying 100–200 bp of each PCR product are listed in Supplementary Table 2. Reactions were carried out for 30 min at 50 °C, followed by 10 min at 95 °C, then followed by 40 cycles of two-step PCR for 15 s at 95 °C, 1 min at 60 °C. The RNA levels of target genes were normalized to *tubulin* RNA and relative concentration was determined using the 2^−ΔΔCt^ Method.

### ROS measurement in vivo

Intracellular ROS levels were determined by measuring the oxidative conversion of cell-permeable 2′,7′-dichlorofluorescein diacetate (DCFH-DA) to fluorescent dichlorofluorescein (DCF) through Reactive Oxygen Species Assay Kit (Beyotime Biotechnology, Shanghai, China). Guts of different developmental host larvae were homogenized in 100 μl NP40 buffer, and centrifuged at 12,000 rpm for 10 min. Then, 10 μl of supernatants were incubated with 100 µmol/L DCFH-DA at 37 °C for 1 h in the dark. Fluorescence was read at 488 nm for excitation and 528 nm for emission with the SpectraMax iD5 Multi-Mode Microplate Reader (Molecular Devices). ROS levels were normalized to the total protein of each sample, which was determined by BCA Protein Assay Kit (Invitrogen). At least three repetitions were performed for each group.

### Immunoblotting

Total protein from fat bodies of *D. melanogaster* 3rd instar larvae was isolated using protein extraction buffer (Solarbio, Beijing, China). After centrifugation at 13,000 rpm for 15 min, supernatants were subjected to SDS-PAGE and transferred to PVDF (Millipore). Membranes were incubated in blocking solution (Tris-buffered saline containing 0.1% Tween 20, 5% BSA) for 1 h. Anti-phospho-AKT (Ser505, Cell Signaling Technology, Danvers, MA), anti-AKT (Cell Signaling Technology, Danvers, MA), and anti-β-tubulin (Beyotime Biotechnology, Shanghai, China) primary antibodies and horseradish-peroxidase-conjugated anti-rabbit IgG and anti-mouse IgG secondary antibody (Solarbio, Beijing, China) were used at a dilution of 1:1000. Resulting immunoblots were visualized using a Bio-Rad Universal Hood II Gel Doc System, while densitometric analysis was performed using Bio-Rad’s Image Lab software.

### *Drosophila* gut microbiota analysis

Host guts were surface sterilized with ethanol and dissected in sterile PBS on an ice plate under a stereoscope (Nikon). A total of 150 guts per experiment and replicate were collected from nonparasitized and parasitized 3rd instar hosts. Bacterial DNA was extracted using the QIAmp DNA Mini Kit (Qiagen). A fragment encompassing the V4 and V5 region of the 16S rRNA gene was amplified using the primers 515F and 926R (Supplementary Table 2). Resulting amplicons were then sequenced on the Illumina MiSeq platform (Shanghai Tiny Gene Bio-Tech Company). FASTA files were filtered to a minimum read length of 200 bp and were clustered into operational taxonomic units (OTUs) at 97% sequence identity using Mothur software (http://www.mothur.org). OTU taxonomies were determined based on the SILVA database (v132, http://www.arb-silva.de) and the National Center for Biotechnology Information 16S rRNA bacterial database as of September 10, 2019 with standard blastn algorithm settings. Gut microbiota experiments were conducted with different *D. melanogaster* host generations: the original generation of both nonparasitized and parasitized larvae (experiment#1) and a second generation (40 generations later) (experiment#2) that included two replicates.

### Isolation of *Acetobacter pomorum*, *Lactobacillus fructivorans* and the other bacteria

Five *D. melanogaster* guts from 3rd instar larvae were homogenized in 200 μl of PBS, and spread onto MRS, LB, and 869 agar plates. 869 agar is a reduced nutrient medium comprised of 0.04 g CaCl_2_–2H_2_O, 0.1 g glucose, D+, 0.5 g NaCl, 1 g tryptone, 0.5 g yeast extract, and 15 g agar in 1000 ml of diluted water. All plates were incubated for 72 h at 30 °C. *A. pomorum*, *L. fructivorans* and the other bacteria were identified by sequencing. Briefly, DNA was isolated from colonies on MRS plates with typical characteristics of *Acetobacter* and *Lactobacillus* bacteria [43] and used as template to PCR amplify products generated using universal 16 S rRNA 27 F and 1522 R primers (Supplementary Table 2). The same DNA isolation and PCR procedures were used for randomly selected colonies on LB agar plates and 869 agar plates. Purified PCR products were Sanger sequenced using an ABI Prism 3730 sequencer (Applied Biosystems). Resulting sequences were then referenced to the GenBank database by BLAST (https://blast.ncbi.nlm.nih.gov/Blast.cgi).

### Quantification of *A. pomorum*

A series of tenfold dilutions of *A. pomorum* were used to construct a standard curve. Briefly, *C*_T_ values in each dilution were measured using qPCR with *A. pomorum-*specific primers. The *C*_T_ values were then plotted against the logarithm of their colony-forming units. Next, the genomic DNA from ten nonparasitized or parasitized *Drosophila* larval guts were extracted using the DNeasy Kit (Qiagen) according to the manufacture protocol. Absolute colony numbers per larval gut were determined from the above standard curves, using the resulting *C*_*T*_ values. qPCR analysis was performed with the AriaMx real-time PCR system (Agilent Technologies) using ChamQ SYBR qPCR Master Mix Kit (Vazyme) and specific primers (Supplementary Table 2).

### Inoculation of AX larvae

Fifteen host guts from 3rd instar nonparasitized CN *Drosophila* larvae were dissected and homogenized in 200 μl PBS. Approximately 50 μl of this gut mixture was added to each axenic food vial containing germ-free embryos. To generate GN *D. melanogaster*, AX larvae were fed *A. pomorum* alone, *L. fructivorans* alone, *A. pomorum* plus *L. fructivorans*, or different combinations of 6 other bacteria identified as gut community members (*M*. sp., *R*. sp., *C*. sp., *B*. sp., *P*. sp., and *Stenotrophomonas* *maltophilia*) (approximately 1 × 10^8^ CFUs for each bacteria). Ten guts for each group were dissected from larvae after inoculation and compared to an AX control by isolating genomic DNA from dissected guts. *A. pomorum* and *L. fructivorans* were then detected by PCR using specific primers (Supplementary Table. 2). Five μl of each PCR reaction was then subjected to agarose gel electrophoresis and visualized. Amplification of the *D. melanogaster tubulin* gene using specific primers (Supplementary Table. 2) served as an endogenous control.

### Statistical analyses

Statistical analyses were performed in GraphPad Prism version 9.0 (GraphPad Software) and SPSS version 19.0 (IBM).

## Results

### Conventional *D. melanogaster* larvae with a gut microbiota support the development of Lb into adults but axenic larvae do not

**Fig. 1:**
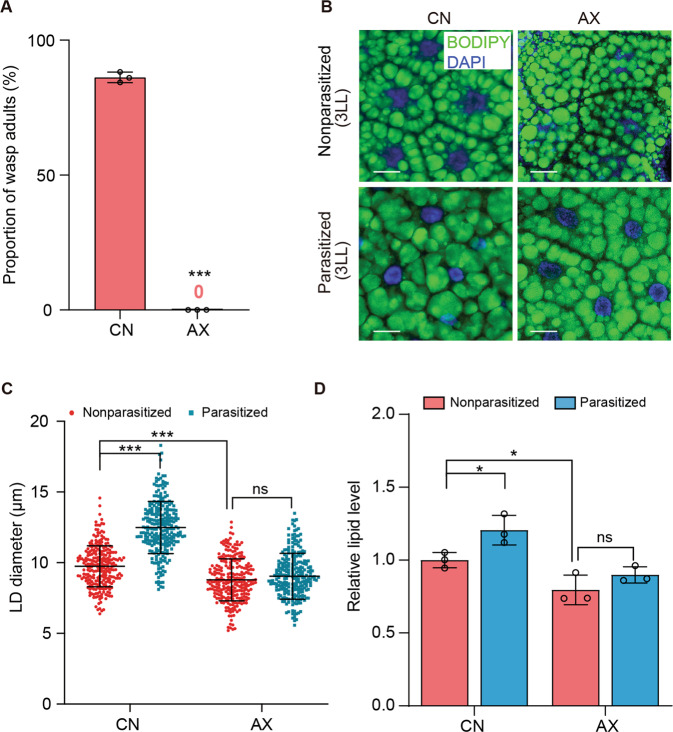
Lb larvae do not develop into adults in AX hosts. **A** The percentage of CN and AX hosts that produce adult Lb. Bars show the mean percentage of hosts ± SD that produced adult Lb in three replicates of 200 hosts for each treatment. Significance was determined by two-sided Mann–Whitney *U* test (***p < 0.001). **B** Fluorescent images of fat body cells from late 3rd instar (3LL) CN or AX larvae that were nonparasitized or parasitized. LDs in fat body cells were labeled by BODIPY (green) while nuclei were labeled by DAPI (blue). Scale bars: 20 μm. **C** LD diameters in (**B**). Each data point is a single LD. A minimum of 200 LDs were measured in fat body cells from 30 individual larvae for each treatment. Error bars show the mean ± SD for each treatment. Significance was determined by two-sided unpaired Student’s *t* test (ns non-significant; ***p < 0.001). **D** Relative lipid levels in fat bodies from 3LL CN and AX larvae that were nonparasitized or parasitized. Fat bodies from 20 individual larvae were measured per replicate. Bars show mean lipid level ± SD from three independent samples. Significance was determined by two-sided unpaired Student’s *t* test (ns: non-significant; *p < 0.05).

Under nutrient-limiting conditions, the gut microbiota promotes the growth of *D. melanogaster* larvae, whereas AX and CN larvae usually exhibit no growth differences when fed nutrient-rich diets [40]. We similarly detected no differences between AX and CN larvae from our laboratory culture in growth to the adult stage when fed a nutrient-rich yeast-based diet (See methods). In contrast, adult Lb emerged from 85% of parasitized CN hosts while no adults emerged from parasitized AX hosts (Fig. 1A). Dissection of host larvae indicated that Lb females parasitized the same proportion of CN and AX hosts as measured by the presence of a wasp egg after oviposition (Supplementary Fig. 2). Lb eggs similarly hatched and developed to the 3rd instar in CN and AX host larvae, which coincided with when hosts ceased feeding as late third instars and initiated metamorphosis to the pupal stage (Supplementary Fig. 3). The fat body in *D. melanogaster* dissociates into individual adipocytes after pupation that are refractive to autophagic cell death, which eliminates most other larval cell types [44]. We observed that the fat body in CN and AX hosts similarly dissociated into individual adipocytes if parasitized by Lb (Supplementary Fig. 3). We also noted that Lb larvae rapidly consumed these dissociated adipocytes, which were visualized by BODIPY which stains neutral lipids in LDs (Supplementary Fig. 3). However, while most Lb larvae in CN host pupae molted to the 5th instar, pupated and emerged as adult wasps, nearly all Lb larvae in AX host pupae remained 4th instars that ultimately died (Fig. 1A, Supplementary Figs. 3 and 4). These observations overall indicated that Lb larvae in both CN and AX hosts consumed adipocytes, but while the former successfully developed into adult wasps the latter did not.

Given the preceding results, we BODIPY stained adipocytes in the fat body of *Drosophila* hosts from the 2nd until the end of the 3rd instar when feeding ceased (Fig. 1B, Supplementary Fig. 5A). This showed that LD diameters increased more in parasitized than nonparasitized CN hosts, which correlated with higher TAG levels (Fig. 1C, D, Supplementary Fig. 5A–C). In contrast, no differences were detected in LD diameters or TAG levels between parasitized and nonparasitized AX hosts, which indicated increased TAG levels after parasitism depended on the presence of a gut microbiota (Fig. 1B–D). We initially thought parasitized CN larvae might have larger lipid stores because they fed more, but bioassays using dye-marked food indicated they overall fed less than nonparasitized CN larvae (Supplementary Fig. 6A, B). We also immunocytochemically visualized neuropeptide F (NPF) and short neuropeptide F (sNPF) in the brains of 3rd instars since both have functions in regulating feeding by *Drosophila* larvae [45, 46]. We detected less NPF in the brains of parasitized than nonparasitized CN larvae (Supplementary Fig. 6C, D), which suggested reduced feeding could be related to downregulation of this neuropeptide. In contrast, we detected no differences in sNPF between parasitized and nonparasitized larvae (Supplementary Fig. 6E, F).

### Lb infection changes the composition of the gut microbiota in host larvae

Recent studies show that lipid metabolic homeostasis in many organisms relies on a healthy commensal community [47, 48]. We thus generated 16S rRNA sequencing libraries from two generations of CN larvae (experiment#1 and experiment#2) to characterize the bacteria present in the guts of *D. melanogaster* third instars (nonparasitized and parasitized) from our laboratory culture (See “Methods”). For the first generation (experiment#1), more than 99% of resulting reads after quality filtering were bacteria in four Phyla (Proteobacteria, Firmicutes, Bacteroidetes, and Actinobacteria) (Supplementary Fig. 7A, Supplementary Table 3). Reads corresponding to Proteobacteria were much lower in the second generation (experiment#2), whereas the abundance of Firmicutes, Fusobacteria, and Epsilonbacteraeota were higher (Supplementary Fig. 7A, Supplementary Table 4). Overall, 58 genera were shared across both generations and replicates, which accounted for 38.80%, 40.75%, and 44.76% of the total reads from experiment#1, experiment#2 replicate 1, and experiment#2 replicate 2 (Supplementary Fig. 7B, Supplementary Tables 3 and 4). At the generic level, only *Lactobacillus* and *Acetobacter* were among the top nine most abundant taxa in each generation and replicate, which was consistent with detection of these genera in many *D. melanogaster* laboratory cultures [49, 50]. High variation in gut microbiota composition has also been reported in other studies where larvae were maintained in the same laboratory and fed the same diet [51, 52]. Our results thus supported a growing consensus that gut microbiota composition is variable due to colonization by a number of opportunistic bacteria, while only a few genera are commonly shared [49, 53]. Gut homogenates plated on MRS agar from experiment#1 yielded a large number of colonies with characteristics of *Acetobacter* and *Lactobacillus* spp. 16 S ribosomal gene sequencing of 30 *Lactobacillus* colonies indicated each was a >99% nucleotide match to *L. fructivorans* (Supplementary Fig. 8), while sequencing of 50 *Acetobacter* colonies indicated each was a > 99% nucleotide match to *Acetobacter pomorum* (Supplementary Fig. 9). Comparing the gut microbiota present in parasitized and nonparasitized CN larvae at the level of genus indicated the most abundant taxa in each treatment was qualitatively similar for experiment #1 and experiment #2 (Supplementary Fig. 7A–C, Supplementary Tables 3–6) although some quantitative differences were also noted including a more than 20-fold increase in *A. pomorum* in parasitized larvae from experiment #1, and a more than 10-fold increase across the two replicates in experiment #2 (Supplementary Fig. 7C). qPCR assays indicated that *A. pomorum* also progressively increased in abundance from the early to late third instar in both nonparasitized and parasitized larvae (Supplementary Fig. 7D).

A major modulator of gut immunity and microbial populations in *Drosophila* is the NADPH oxidase *Duox*, which can generate reactive oxygen species (ROS) to defend against pathogenic bacteria [39, 54]. To test whether changes in *Duox* expression might underlie the microbiome changes induced by parasitoid infection, we used RT-qPCR to measure relative *Duox* RNA. Expression of *Duox* was significantly reduced in the guts of parasitized *Drosophila* larvae, while *Duox* expression in carcasses lacking the guts remained unaltered (Supplementary Fig. 7E). We also measured ROS levels using 2′,7′-dichlorofluorescein diacetate (DCFH-DA) and found a significant reduction in the guts in response to parasitization (Supplementary Fig. 10). Bacteria in the guts can also be controlled by antimicrobial peptides (AMPs) [55]. We tested the expression profiles of six AMP genes, chosen based on their roles in *Drosophila* gut immunity. Interestingly, three AMP genes encoding *Diptericin* (*Dpt*), *Attacin* (*Att*), and *Metch* were significantly up-regulated in the guts after parasitization (Supplementary Fig. 7F), and all six AMP genes were up-regulated in carcasses lacking the guts (Supplementary Fig. 7G). Thus, parasitism strongly affected the expression of Duox and AMPs, two major classes of bactericidal agents in the guts, and substantially altered the host gut microbiota population including a large increase in the proportion of *A. pomorum*.

### GN larvae inoculated with certain gut community members support Lb development

**Fig. 2:**
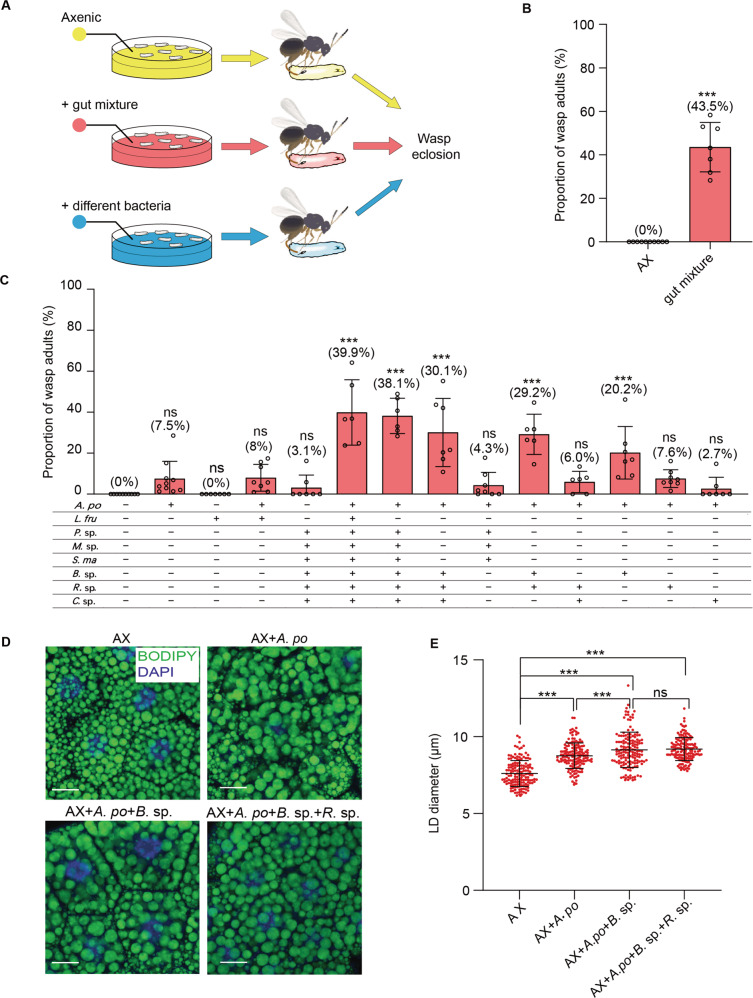
GN larvae with *A. pomorum* and *Bacillus* sp. promote Lb survival. **A** Schematic showing how AX hosts were inoculated with bacteria to produce GN hosts that were parasitized. The percentage of hosts for each treatment producing adult wasps (eclosion) were then determined. **B** Results showing the percentage of AX and GN hosts that produced adult wasps from the experimental design shown in (**A**). Treatments were parasitized AX hosts and GN hosts inoculated with gut mixture from CN larvae. From left to right: *n* = 672, 368. Bars show the mean percentage of hosts ± SD that produced adults in at least six replicates per treatment. Significance was determined by two-sided Mann–Whitney *U* test (***p < 0.001). **C** Results showing the percentage of AX and GN hosts that produced adult wasps from the experimental design shown in (**A**). Treatments were parasitized AX hosts and GN hosts inoculated with *A. pomorum* only*, L. fructivorans* only, *A. pomorum* plus *L. fructivorans*, *A. pomorum* plus combinations of six other bacteria identified as gut community members. From left to right: *n* = 672, 641, 581, 655, 415, 782, 496, 634, 547, 383, 483, 781, 616, 422. *A. po* *Acetobacter pomorum*, *L. fru* *Lactobacillus fructivorans*, *P*. sp. *Pseudomonas* sp., *M*. sp. *Mesorhizobium* sp., *S*. *ma* *Stenotrophomonas maltophilia*, *B*. sp. *Bacillus* sp., *R*. sp. *Rhodococcus* sp., *C*. sp. *Chitinophaga* sp. Bars show the mean percentage of hosts ± SD that produced adult in at least six replicates per treatment. Significance was determined by Kruskal–Wallis test with Dunn’s multiple comparisons test. (ns non-significant; ***p < 0.001). **D** BODIPY and DAPI labeled images of fat body cells from 3LL GN *D. melanogaster* larvae inoculated with *A. pomorum* only, *A. pomorum* and *Bacillus*. sp., or *A. pomorum*, *Bacillus*. sp. and *Rhodococcus* sp. Scale bars: 20 μm. **E** Quantification of LD diameters in (**D**). Each data point is a single LD. A minimum of 150 LDs were measured in fat body cells from 20 individual larvae for each treatment. Error bars show the mean ± SD for each treatment. Significance was determined by Kruskal–Wallis test with Dunn’s multiple comparisons test. (ns: non-significant; ***p < 0.001).

To investigate whether particular members of the host gut microbiota affect development of Lb, we made gut homogenates from nonparasitized CN hosts. We referred to this homogenate as a “gut mixture” that we used to inoculate AX larvae (Fig. 2A). We then allowed Lb females to parasitize these hosts, which resulted in 43.5% yielding adult wasps (Fig. 2B). This outcome indicated a mixed community of microbes from the guts of CN larvae significantly increased wasp survival when compared to AX hosts from which no adult wasps emerge. However, a successful parasitism rate of 43.5% was also lower than ~85% of CN hosts producing Lb adults when the gut microbiota was naturally acquired from the environment (see Fig. 1A). Since *A*. *pomorum* and *L*. *fructivorans* were consistently abundant gut community members, we next assessed whether AX larvae inoculated with either or both of these species produced GN larvae that supported Lb development. Both successfully colonized AX larvae (Supplementary Fig. 11) but only 7.5% of GN larvae inoculated with *A*. *pomorum* and 8.0% of GN larvae inoculated with *A*. *pomorum* and *L*. *fructivorans* produced adult wasps while no GN larvae inoculated with only *L*. *fructivorans* did so (Fig. 2C). We thus isolated other bacteria present in the guts of CN larvae by plating gut homogenates on LB and 869 agar plates. Randomly picking and sequencing 95 colonies from LB plates indicated 40 were a *Mesorhizobium* sp. (Supplementary Fig. 12), 25 were a *Rhodococcus* sp. (Supplementary Fig. 13), 15 were a *Chitinophaga* sp. (Supplementary Fig. 14), 10 were a *Bacillus* sp. (Supplementary Fig. 15), and 5 were a *Pseudomonas* sp. (Supplementary Fig. 16). Doing the same for 152 colonies from 869 agar plates indicated 92 were *A*. *pomorum*, 26 were the *Mesorhizobium* sp., 20 were *S. maltophilia* (Supplementary Fig. 17), and the remaining 14 were the *Pseudomonas* sp. Inoculating AX larvae with a mixture of *S. maltophilia*, *Mesorhizobium* sp., *Rhodococcus* sp., *Chitinophaga* sp., *Bacillus* sp. and *Pseudomonas* sp. resulted in only 3.1% of hosts producing adult Lb (Fig. 2C). However, inoculating larvae with these six species plus *A*. *pomorum* or *A*. *pomorum* and *L*. *fructivorans* resulted in 38-40% of hosts producing Lb which was comparable to inoculating larvae with our gut mixture from CN larvae (Fig. 2C). Inoculating AX larvae with *A*. *pomorum* plus *Bacillus* sp. and *Rhodococcus* sp. resulted in 29.2% of GN larvae producing adult Lb, while AX larvae inoculated with *A*. *pomorum* plus *Bacillus* sp. resulted in 20.2% of GN larvae producing adult Lb (Fig. 2C). Comparing LD sizes among treatments further indicated that AX larvae inoculated with *A*. *pomorum* plus *Bacillus* sp. or *A*. *pomorum* plus *Bacillus* sp. and *Rhodococcus* sp. were larger than AX larvae inoculated with *A*. *pomorum* alone (Fig. 2D, E). Thus, *A*. *pomorum* together with the *Bacillus* sp. alone or in combination with the *Rhodococcus* sp. significantly increased the proportion of GN larvae that produced Lb adults which was associated with increased LD size in fat body.

### Lipid stores in the fat body of parasitized CN hosts are affected by reduction of Bmm lipase activity

**Fig. 3:**
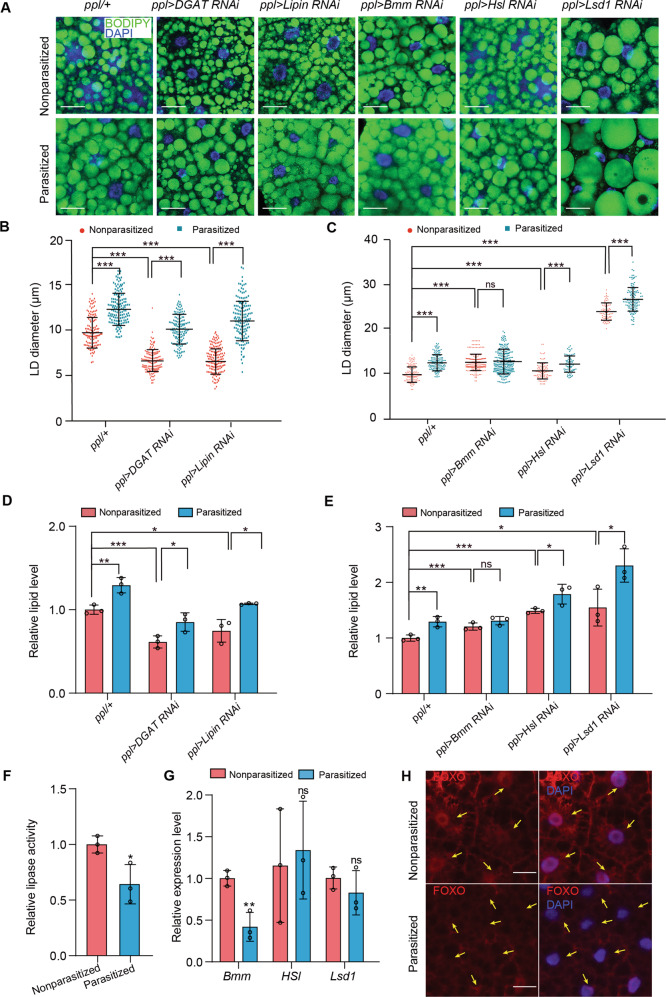
Parasitism reduces lipid mobilization in CN hosts. **A** Fluorescent images of fat bodies from nonparasitized and parasitized hosts. Genotypes are as follows: *ppl/+* (*ppl-Gal4*/+), *ppl* > *DGAT RNAi* (*ppl-Gal4*/+; *UAS-DGAT RNAi*/+), *ppl* > *Lipin RNAi* (*ppl-Gal4*/+; *UAS-Lipin RNAi*/+), *ppl* > *Bmm RNAi* (*ppl-Gal4*/+; *UAS-bmm RNAi*/+), *ppl* > *Hsl RNAi* (*ppl-Gal4*/+; *UAS-Hsl RNAi*/+) and *ppl* > *Lsd1 RNAi* (*ppl-Gal4*/+; *UAS-lsd1 RNAi*/+). LDs were labeled by BODIPY (green) and nuclei were labeled by DAPI (blue). Scale bars: 20 μm. **B, C** LD diameters in (**A**). Each data point is a single LD. A minimum of 200 LDs were measured in fat body cells from 30 individual larvae for each treatment. Error bars show the mean ± SD for each treatment. Significance was determined by two-sided unpaired Student’s *t* test (ns: non-significant; ***p < 0.001). **D** Relative lipid levels in fat bodies from nonparasitized and parasitized larvae *ppl* > *DGAT* or *ppl* > *Lipin RNAi* hosts. Fat bodies from 20 larvae were measured per replicate. Bars show mean lipid level ± SD from three independent samples. Significance was determined by two-sided unpaired Student’s *t* test (*p < 0.05; **p < 0.01; ***p < 0.001). **E** Relative lipid levels in fat bodies from nonparasitized and parasitized *ppl* > *Bmm, ppl* > *Hsl*, or *ppl* > *Lsd1 RNAi* hosts. Fat bodies from 20 larvae were measured per replicate. Bars show mean lipid level ± SD from three independent samples. Significance was determined by two-sided unpaired Student’s *t* test (ns non-significant; *p < 0.05; **p < 0.01; ***p < 0.001). **F** Relative lipase activity in fat bodies from 3rd instar nonparasitized and parasitized hosts (*n* = 20 pooled larvae per sample). Bars for each treatment show the mean ± SD from three independent samples. Significance was determined by two-sided unpaired Student’s *t* test (*p < 0.05). **G** Relative mRNA levels of *Bmm*, *Hsl*, and *Lsd1* in fat bodies from 3rd instar nonparasitized and parasitized hosts (*n* = 10 pooled larvae per sample). Bars for each treatment show the mean ± SD from three independent experiments. Significance was determined by two-sided unpaired Student’s *t* test (ns: non-significant; **p < 0.001). **H** Immunocytochemical localization of FOXO by an anti-FOXO antibody (red) in larval fat body cells. FOXO is detected in the cytoplasm and nuclei of cells from nonparasitized hosts, whereas FOXO localization was reduced in nuclei from parasitized hosts (arrowheads). Nuclei were labeled by DAPI (blue). Scale bar: 50 μm.

Four enzymatic steps regulate the conversion of glycerol-3-phosphate to TAG by esterification with fatty acids [32]. In *Drosophila*, Lipin converts phosphatidic acid into diacylglycerol (DAG) in the third step, while diacylglycerol acyltransferase (DGAT) converts DAG to TAG in the fourth step (Supplementary Fig. 18) [56, 57]. In turn, lipolysis involves two lipases, Bmm and hormone-sensitive Hsl, plus lipid storage droplet protein 1 (Lsd1) that recruits Hsl but not Bmm to the surface of LDs (Supplementary Fig. 18) [58]. To investigate the direct effectors of parasitization-induced lipid accumulation, we used the *ppl-gal4* driver to knock down lipogenesis and lipolysis genes in the fat body. We found that fat bodies with reduced expression of the lipogenesis genes *Lipin* or *DGAT* had smaller LDs and neutral lipids stores in nonparasitized CN larvae (Fig. 3A, B, D), confirming an important role of Lipin and DGAT in TAG synthesis in vivo. Nevertheless, parasitism by Lb still increased LD size and TAG levels in the hosts with reduced Lipin or DGAT activity in CN larvae (Fig. 3A, B, D). Thus, Lb greatly alters LD size under conditions of severely reduced lipogenic capacity, suggesting that parasitism primarily alters host lipolysis rather than lipogenesis. As expected, knockdown of *Bmm* or *Hsl* or *Lsd1* increased lipid stores in nonparasitized CN larvae (Fig. 3A, C, E). LD size and TAG level were further increased upon parasitization in Hsl-deficient CN larvae, Lsd1-deficient CN larvae but not in Bmm-deficient CN larvae, suggesting that parasitism increases lipid stores in host fat body principally by reducing Bmm lipase activity (Fig. 3A, C, E). Consistent with this hypothesis and a key role for regulating lipolysis, lipase activity in the fat body was also overall lower in parasitized versus nonparasitized CN larvae which correlated with lower transcript levels for *Bmm* but not *Hsl* or *Lsd1* (Fig. 3F, G). Transcription of *Bmm* depends on FOXO activity in the nucleus, which functions as a regulatory intermediate of PI3K-AKT signaling through the IIS pathway [33, 59]. As such, increased insulin signaling in parasitized CN larvae is thought to reduce *Bmm* gene expression through phosphorylation and associated inhibition of FOXO entry into the nucleus [32, 60], which was consistent with our results showing that nuclear FOXO was lower in parasitized than nonparasitized CN larvae (Fig. 3H). Collectively then, the preceding results indicated parasitism enhanced lipid accumulation by down-regulating FOXO nuclear activity and which in turn reduced Bmm lipase activity in the fat body of CN hosts.

Since knockdown of DGAT reduced lipogenesis as measured by smaller lipid stores, we asked if Lb development was adversely affected in parasitized hosts where DGAT was knocked down. Results showed that Lb usually developed to the adult stage in DGAT knockdown hosts but males showed a 1-day delay in emergence while females showed a 2-day delay (Supplementary Fig. 19A, B). Adult female wasps were also smaller than female wasps from control hosts (Supplementary Fig. 19C). Since parasitism depends on adult females injecting both eggs and venom secretions into hosts, we compared ovary and venom gland sizes between females that emerged from DGAT knockdown and control hosts. Venom gland but not ovary size was significantly smaller in Lb females from DGAT knockdown hosts (Supplementary Fig. 19D-G). The reduction in venom gland size led us to hypothesize that females from DGAT knockdown hosts could also suffer a fitness penalty in parasitizing new hosts. Indeed, rates of successful parasitism were lower for females that emerged from DGAT knockdown than control hosts (Supplementary Fig. 19H). Taken together, knockdown of DGAT resulted in lipid stores that were lower than in control parasitized hosts but were higher than in parasitized AX hosts. Correspondingly, Lb fitness was reduced in parasitized CN hosts where DGAT was knocked down when compared to control CN hosts, but was also much higher than in AX hosts where Lb failed to develop into adults.

### Elevated insulin signaling promotes lipid accumulation in parasitized hosts with a gut microbiota

**Fig. 4:**
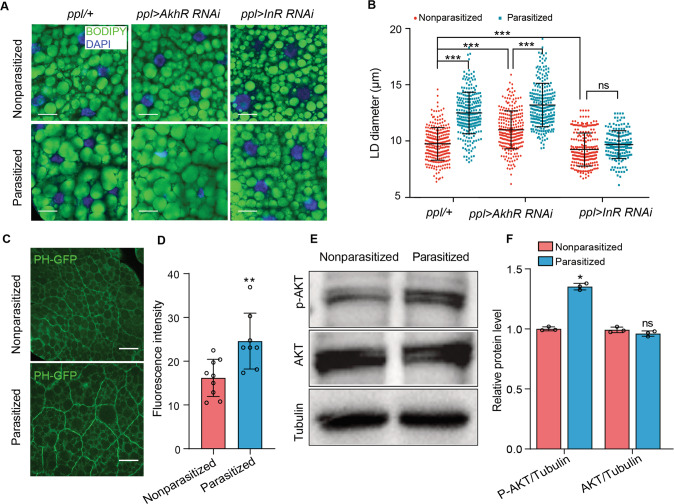
Increased LD size is associated with elevated insulin signaling. **A** Fat body cells from nonparasitized and parasitized CN hosts with LDs labeled with BODIPY (green) and nuclei labeled with DAPI (blue). Genotypes are: *ppl/+* (*ppl-Gal4*/+), *ppl* > *AkhR RNAi* (*ppl-Gal4*/+; *UAS-AkhR RNAi*/+), and *ppl* > *InR RNAi* (*ppl-Gal4*/+; *UAS-InR RNAi*/+). Scale bars: 20 μm. **B** LD diameters in (**A**). Each data point is a single LD. A minimum of 200 LDs were measured in fat body cells from 30 individual larvae for each treatment. Error bars show the mean ± SD for each treatment. Significance was determined by two-sided unpaired Student’s *t* test (ns: non-significant; ***p < 0.001). **C** PI3K activity in nonparasitized and parasitized fat body cells as marked by PH-GFP (green). Scale bars: 50 μm. **D** Fluorescence intensity of PH-GFP on the fat body membrane of nonparasitized and parasitized hosts. At least 100 fat body cells from 30 individual larvae were measured per treatment. Bars show mean fluorescence intensity per cell ± SD. Significance was determined by two-sided unpaired Student’s *t* test (**p < 0.01). **E**, **F** Immunoblot analysis of phosphorylated (pS505) AKT, total AKT, and β-tubulin (loading control) in the fat body of nonparasitized and parasitized host larvae. p-AKT and AKT levels were normalized to the loading control in comparing relative protein levels. Bars show mean ± SD from three immunoblots prepared from independent samples. Significance was determined by two-sided unpaired Student’s *t* test (ns: non-significant; *p < 0.05).

Since *Drosophila* adipokinetic hormone (AKH) and insulin-like peptides (Dilps) are key regulators of lipid homeostasis [61], we used the *ppl-Gal4* driver to knock down the AKH (*UAS-AkhR RNAi*) and insulin-like receptor (*UAS-InR RNAi*) in the fat body [38]. Depletion of the AkhR in nonparasitized CN third instars increased LD diameter when compared to nonparasitized control larvae (Fig. 4A, B). This outcome was consistent with *AkhR* null mutants promoting obesity [61] and overexpression of *AKH* or *AkhR* reducing fat stores [35]. However, *AkhR* depletion in parasitized CN larvae increased LD diameters more than in nonparasitized CN larvae, which suggested the larger lipid stores in parasitized hosts did not require the AkhR. In contrast, *InR* depletion reduced LD diameters in both nonparasitized and parasitized CN larvae (Fig. 4A, B), while activation of the insulin pathway via reduction of Pten (phosphatase and tensin homolog) increased LD diameters in nonparasitized CN larvae to similar levels as parasitized CN larvae (Supplementary Fig. 20A, B). *Tub-PH-GFP* encodes the pleckstrin homology (PH) domain of the *Drosophila* Steppke (Grp1) protein which is fused to GFP under control of the *tubulin* gene promoter. This results in global expression at a modest level and recruitment of the GFP-tagged PH domain to the surface of cells via binding to phosphatidylinositol 3, 4, 5-trisphosphate (PIP3), which is the second messenger generated by phosphoinositide 3-kinase (PI3K). The GFP signal thus provides a measure of insulin pathway signaling via PI3 kinase activity [62]. PH-GFP levels in fat body adipocytes were higher in parasitized than nonparasitized CN larvae (third instars) (Fig. 4C, D). Increased PIP3 levels further result in activation of protein kinase AKT, a central target of the InR-PI3K pathway, via phosphorylation at residue Ser505 [63]. Immunoblotting assays showed that Ser505 phosphorylation in fat body extracts was also higher in parasitized than nonparasitized CN larvae (Fig. 4E, F).

**Fig. 5:**
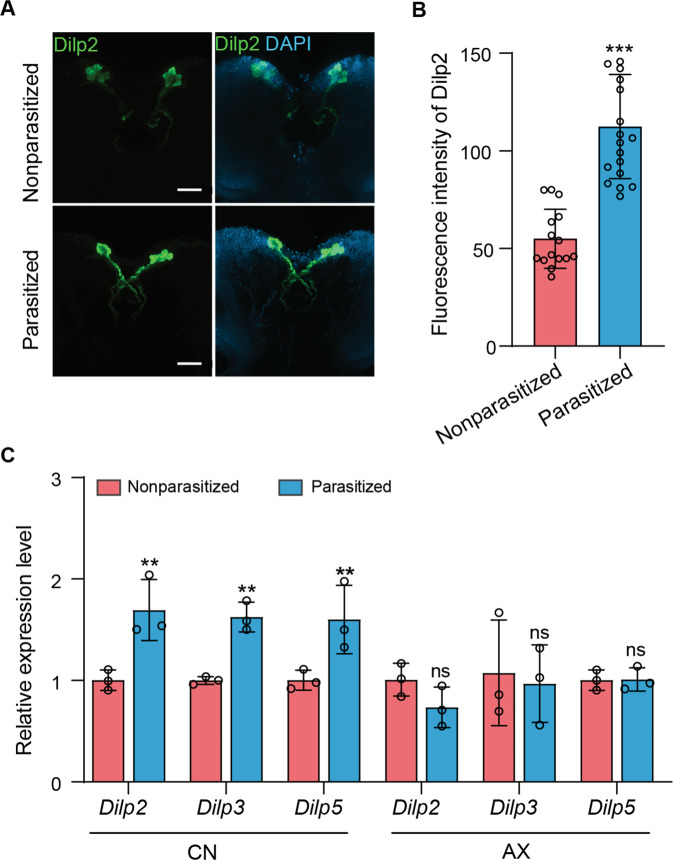
Dilps are overexpressed in parasitized CN hosts. **A** Immunohistochemical analysis of Dilp2 in IPCs from nonparasitized and parasitized hosts. IPCs were labeled with anti-Dilp2 (green) while nuclei were labeled with DAPI (blue). Scale bars: 100 μm. **B** Fluorescence intensity of Dilp2 in IPCs from nonparasitized and parasitized hosts (*n* = 20 for each group). Bars show mean fluorescence per cell ± SD. Significance was determined by two-sided unpaired Student’s *t* test (***p < 0.001). **C** Relative mRNA levels of *Dilp2*, *Dilp3*, and *Dilp5* in brains from CN and AX 3rd instar hosts that were nonparasitized or parasitized (*n* = 20 for each group). Bars show mean ± SD from three independent experiments. Significance was determined by two-sided unpaired Student’s *t* test (ns: non-significant; **p < 0.01).

The preceding results indicated that parasitism of CN hosts increases insulin signaling above what normally occurs in nonparasitized CN hosts. Three (*Dilp2*, *Dilp3* and *Dilp5*) of the eight *Dilp* genes are selectively expressed in ilp-producing medial neurosecretory cells (IPCs) and are known regulators of metabolic homeostasis in the fat body [64]. Dilp2 was more strongly detected in IPCs from parasitized than nonparasitized CN larvae by anti-Dilp2 (Fig. 5A, B), as was a *Dilp2-Gal4* transgene that drove *UAS-GFP* expression (Supplementary Fig. 21). RT-qPCR assays also detected higher expression of *Dilp2*, *Dilp3* and *Dilp5* in the brains of parasitized than nonparasitized CN larvae (Fig. 5C). In contrast, no differences were detected in the expression of *Dilp* *2*, *3* and *5* between parasitized and nonparasitized AX larvae, which strongly suggested elevated expression required the presence of a gut microbiota (Fig. 5C).

## Discussion

**Fig. 6:**
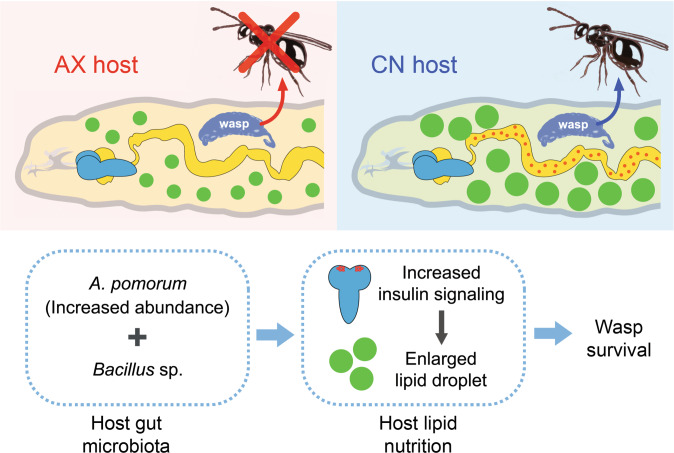
The proposed model of how host gut microbiota promotes parasite survival. Lb successfully develops into adults in conventional hosts (CN) but fails to develop in axenic hosts (AX). In addition, increased abundance of *A. pomorum* plus other identified gut community members including a *Bacillus* sp. are necessary for wasp survival, which is associated with increased insulin signaling in brain neurosecretory cells of parasitized CN larvae and increased lipid accumulation in host fat body cells.

Host nutrients are a key currency that parasites often manipulate [1, 5, 18], while the gut microbiota of many host organisms influences metabolism or susceptibility to infection [2, 47]. Here, we present results where these inter-species interactions intersect: a parasite that increases host lipid stores for nutritional needs and survival but also requires the host gut microbiota to do so (Fig. 6).

Lipid profiles are probably the best-characterized parasitism-associated metabolic alteration [20, 25, 26]. Host lipid profiles are also known to affect the metabolism, survival and/or transmission of some parasites [1, 16]. For example, *Toxoplasma gondii* secures cellular fatty acids essential for its proliferation [20]. While parasitoid wasps are unable to *de novo* synthesize lipids [15] and thus rely on host lipid metabolism to meet dietary needs for development into adults [21, 23, 65]. In this study, we identify a developmental strategy whereby Lb allows the host to grow to the pupal stage, but its development into adults also depends on increased lipid accumulation in fat body cells of hosts that wasp larvae consume. Our results indicate increased lipid accumulation involves increased expression of *Dilps* in IPCs and insulin signaling in the host fat body. How Lb stimulates this increase in insulin signaling remains unclear but our results do reveal the novel finding that manipulation of host metabolism by the wasp depends on the presence of a gut microbiota in the host. Key evidence supporting this conclusion is our finding that AX larvae inoculated with gut microbes from CN larvae substantially increases the proportion of parasitized hosts that produce adult Lb. While *A. pomorum* has previously been reported to positively modulate *Drosophila* energy metabolism by producing modulators like acetic acid that promote insulin signaling [47], inoculating AX larvae with this gut community member alone or *L. frutivorans* does not substantially increase the proportion of parasitized GN larvae that produce adult Lb. In contrast, inoculating AX larvae with *A. pomorum* plus six other gut community members increases the proportion of Lb that develop into adults to similar levels as our positive control (gut mixture). *A. pomorum* plus a *Bacillus* sp. present in CN larvae also substantially increased the proportion of GN larvae that produce adult Lb. We thus conclude that successful development of Lb depends on particular members of the gut microbiota present in CN larvae. A key goal going forward is to identify how the gut microbiota promotes insulin signaling and nutrient storage in the fat body even though parasitized host larvae feed less than nonparasitized larvae. Another is to determine how gut community members interact to promote Lb fitness given that *A. pomorum* plus six other gut community members or *A. pomorum* plus *Bacillus* sp. in GN larvae substantially increase the proportion of Lb larvae that develop into adults but do not restore survival to levels in CN larvae that acquire a gut microbiota by feeding. Our results overall suggest LD quantity in host adipocytes does affect Lb development or adult fitness. However, our finding that hosts where DGAT was knocked down produced adult wasps with reduced fitness while AX hosts produced no adult wasps also suggests the host gut microbiota potentially affects the specific types of lipids that are stored in the fat body which in turn influence Lb development. Thus, profiling lipid composition in parasitized versus nonparasitized hosts and how lipid composition is potentially affected by specific gut community members is another important goal for future investigation.

In summary, our results demonstrate reliance of a parasite on the gut microbiota of a host for nutritional needs and survival. Our results also provide new insights into the mechanisms underlying how Lb manipulates the metabolism of hosts with a gut microbiota. Parasitoid wasps are extremely diverse with more than 150,000 described species and estimates that suggest ~20% of all insects are parasitoids [66, 67]. Given the enormous number of wasp species with life histories that are similar to Lb, our findings point to the possible common strategy by which many parasitoids interact with the host gut microbiota to meet nutritional requirements that enhance their fitness. Our findings further suggest the possibility that other parasites including species that cause disease in humans and other vertebrates are also potentially affected by the gut microbiota of their hosts.

## Supplementary information

Supplementary information

Supplementary Tables S3 to S6

## Data Availability

16S rRNA gene sequence data have been deposited in the National Center for Biotechnology Information Sequence Read Archive (accession no. PRJNA550325 for experiment#1 and PRJNA679648 for experiment#2). All other data are available in the main text or Supplementary materials.
