# Supplementary material for: Parasite reliance on its host gut microbiota for nutrition and survival
Source: ISME J. 2022 Aug 8;16(11):2574–86. doi: 10.1038/s41396-022-01301-z (PMC9561699; doi:10.1038/s41396-022-01301-z)
Supplement: Supplementary file 1 — Supplementary information [file 41396_2022_1301_MOESM1_ESM.docx]

**Supplementary Information**

**Parasite reliance on its host gut microbiota for nutrition and survival**

Sicong Zhou^1,2,3§^, Yueqi Lu^1,2,3§^, Jiani Chen^1,2,3^, Zhongqiu Pan^1,2,3^, Lan Pang^1,2,3^, Ying Wang^1,2,3^, Qichao Zhang^1,2,3^, Michael R. Strand^4,*^, Xue-Xin Chen^1,2,3,5,*^, Jianhua Huang^1,2,3,*^

^1^Institute of Insect Science, College of Agriculture and Biotechnology, Zhejiang University, Hangzhou 310058, China

^2^Ministry of Agriculture Key Lab of Molecular Biology of Crop Pathogens and Insect Pests, Zhejiang University, Hangzhou 310058, China

^3^Key Laboratory of Biology of Crop Pathogens and Insects of Zhejiang Province, Zhejiang University, Hangzhou 310058, China

^4^Department of Entomology, University of Georgia, Athens, GA 30602, USA

^5^State Key Lab of Rice Biology, Zhejiang University, Hangzhou 310058, China

^§^These authors contributed equally

^*^Corresponding authors. [jhhuang@zju.edu.cn](mailto:jhhuang@zju.edu.cn); xxchen@zju.edu.cn; mrstrand@uga.edu

This PDF file includes:

Figures S1 to S21

Tables S1 and S2

**Other Supplementary Materials for this manuscript include the following:**

Tables S3-S6 are presented in a separate Excel file due to size.

Legends for Table S3-S6.

- - Table S3. Relative abundance of Operational Taxonomic Units (OTUs) in guts from nonparasitized *Drosophila* 3^rd^ instar larvae in experiment#1.
  - Table S4. Relative abundance of Operational Taxonomic Units (OTUs) in guts from nonparasitized *Drosophila* 3^rd^ instar larvae in experiment #2.
  - Table S5. Relative abundance of Operational Taxonomic Units (OTUs) in guts from parasitized *Drosophila* 3^rd^ instar larvae in experiment#1.
  - Table S6. Relative abundance of Operational Taxonomic Units (OTUs) in guts from parasitized *Drosophila* 3^rd^ instar larvae in experiment#2.

**
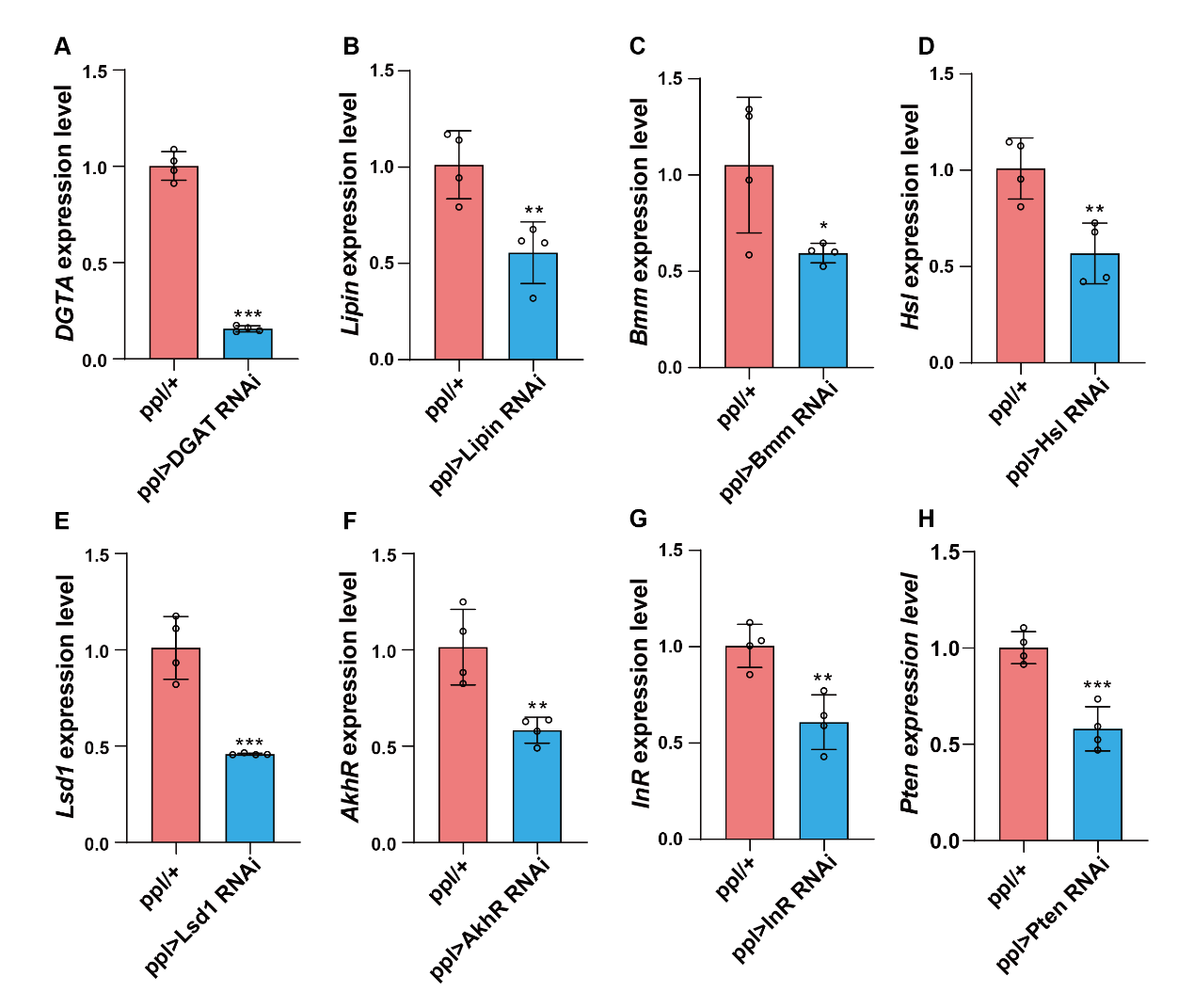
**

**Suppl. Fig. 1. Validation the knockdown efficiency of the RNAi stocks.**

Relative mRNA levels of the tested RNAi genes: *DGAT* (**A**), *Lipin* (**B**), *Bmm* (**C**), *Hsl* (**D**), *Lsd1* (**E**), *AkhR* (**F**), *InR* (**G**), and *Pten* (**H**) in host fat body cells. Error bars show the mean ± SD from four independent replicates. Significance was determined by two-sided unpaired Student’s *t* test (*: *p* < 0.05; **: *p* < 0.01; ***: *p* < 0.001).


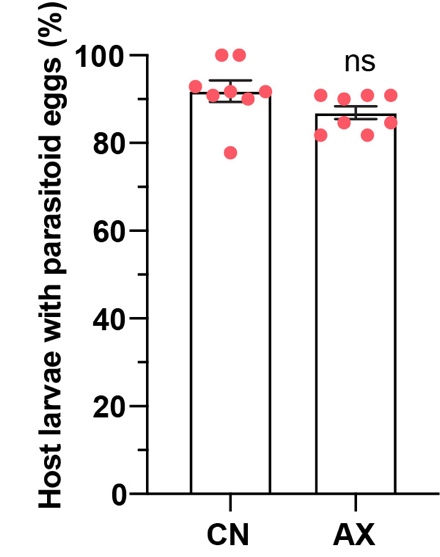


**Suppl. Fig. 2. Wasp oviposition performance in CN and AX hosts.**

The percentage of CN and AX hosts that contained parasitoid eggs after Lb females were allowed to oviposit (n = 80 for each group)*.* Bars show the mean ± SD of eight independent replicates. Significance was determined by two-sided unpaired Student’s *t* test (ns: non-significant), while the red dots indicate the percentage of hosts in each replicate that contained a wasp egg. The female wasps were allowed to parasitize the hosts for 3 h at a wasp/host ratio of 1:10, then host larvae were dissected from each replicate.

**
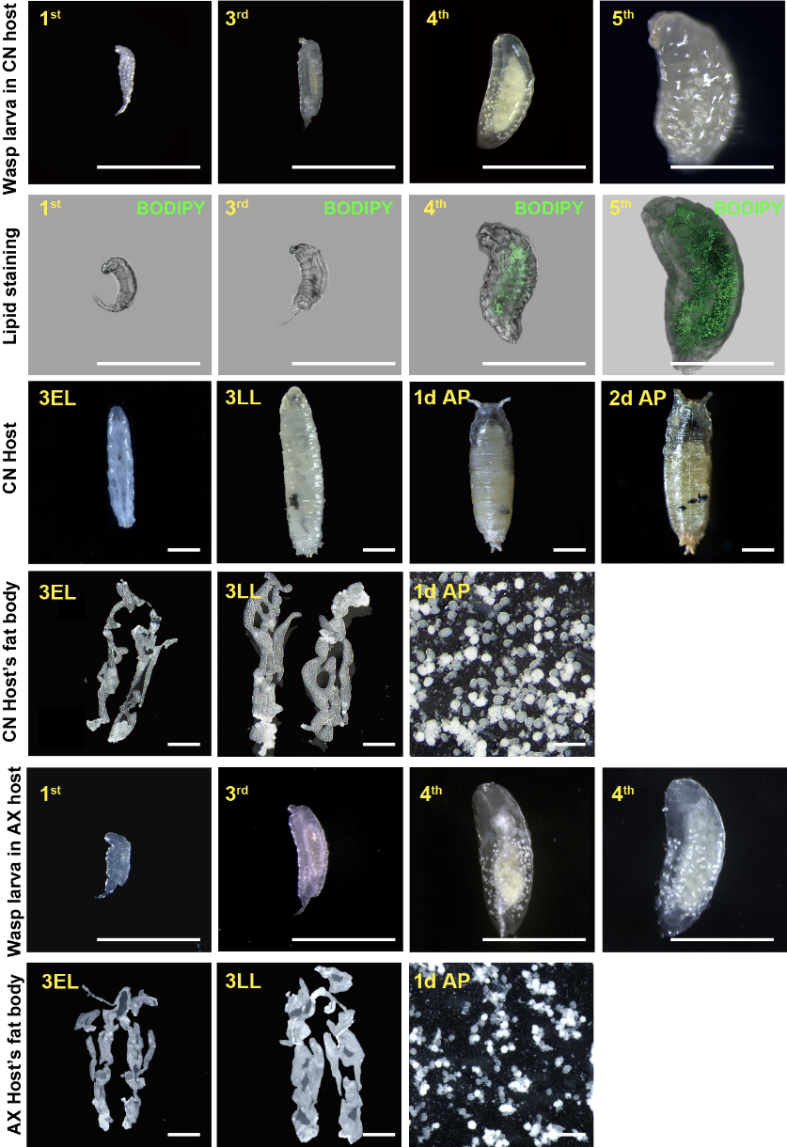
**

**Suppl. Fig. 3. Micrographs showing host stages, host fat body, and development of Lb larvae.**

Wasps lay their eggs in 2^nd^ instar *D. melanogaster* larvae, eggs hatch into 1^st^ instar larvae in 3EL hosts, and molt to the 3^rd^ instar in 3LL hosts. When CN hosts pupate, wasp larvae molt to the 4^th^ instar 1d AP and to the 5^th^ instar 2 d AP. Spherical, free-floating fat body cells are present at 1d APF in both CN and AX hosts. Wasps consume host fat body cells after molting to the 4^th^ instar. In CN hosts, wasp larvae consume all available host fat body cells after molting to the 5^th^ instar. Lipids in the guts of 4^th^ and 5^th^ instar wasp larvae (green) were stained by BODIPY. However, in AX hosts, wasps remain in the 4^th^ instar in 2 d AP AX hosts. Scale bars: 200 μm for images of wasp larvae, 500 μm for images of *Drosophila* larvae, pupae and fat body. Host stages: Early 3^rd^ instar Larvae; 3LL: Late 3^rd^ instar Larvae; 1d AP: 1 day after host pupation; 2d AP: 2 days after host pupation.


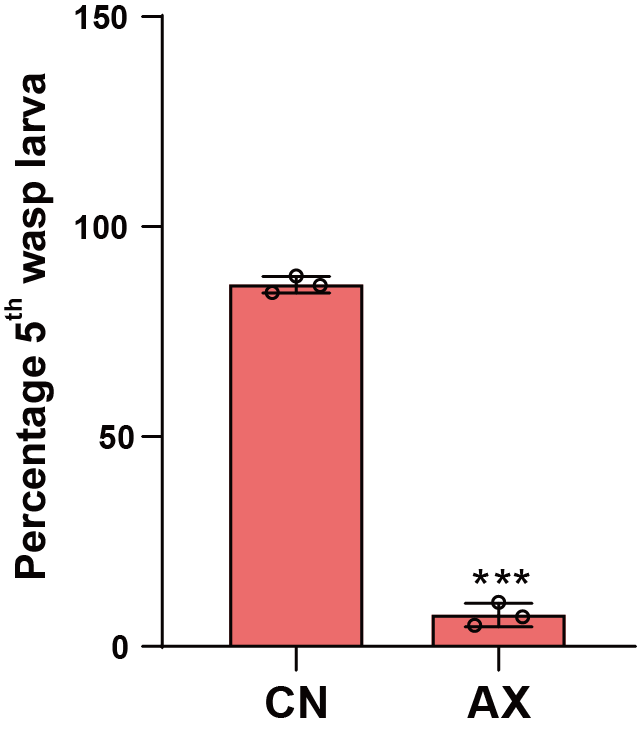


**Suppl. Fig. 4. Most 4^th^ instar wasp larvae do not molt to the 5^th^ instar in AX hosts.** n = 200 for each group*.* Bars show represent mean ± SD of three independent experiments. Significance was determined by two-sided unpaired Student’s *t* test (***: *p* < 0.001).


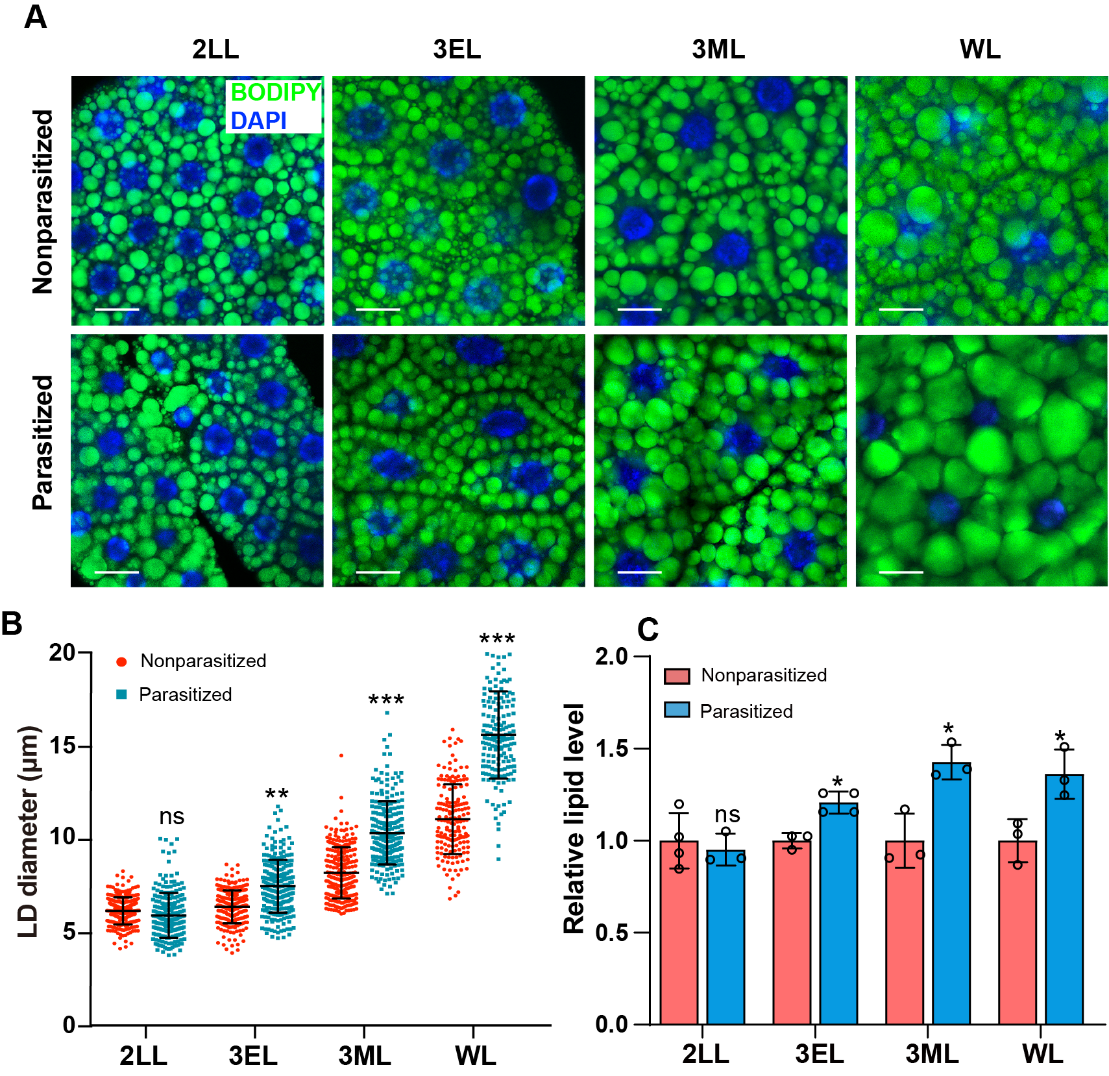


**Suppl. Fig. 5. Parasitism promotes lipid accumulation in the host fat body.**

**A** Fluorescent images of fat body cells from non-parasitized and parasitized 2LL, 3EL, 3ML, and WL host larvae. LDs are labeled by BODIPY (green) and nuclei are labeled by DAPI (blue). Scale bars: 20 μm.

**B** Quantification of LD diameters in (A). Each data point is a single LD. A minimum of 200 LDs were measured in fat body cells from 30 individual larvae for each treatment. Error bars show the mean ± SD for each treatment. Significance was determined by two-sided unpaired Student’s *t* test (ns: non-significant; **: *p* < 0.01; ***: *p* < 0.001).

**C** Relative TAG levels in fat bodies from non-parasitized and parasitized larvae from different developmental stages (n=20 for each group). Bars show mean ± SD of three independent experiments. Significance was determined by two-sided unpaired Student’s *t* test (ns: non-significant; *: *p* < 0.05). 2LL: Late 2^nd^ instar Larvae; 3EL: Early 3^rd^ instar Larvae; 3ML: Middle 3^rd^ instar Larvae; WL: Wandering Larvae.**
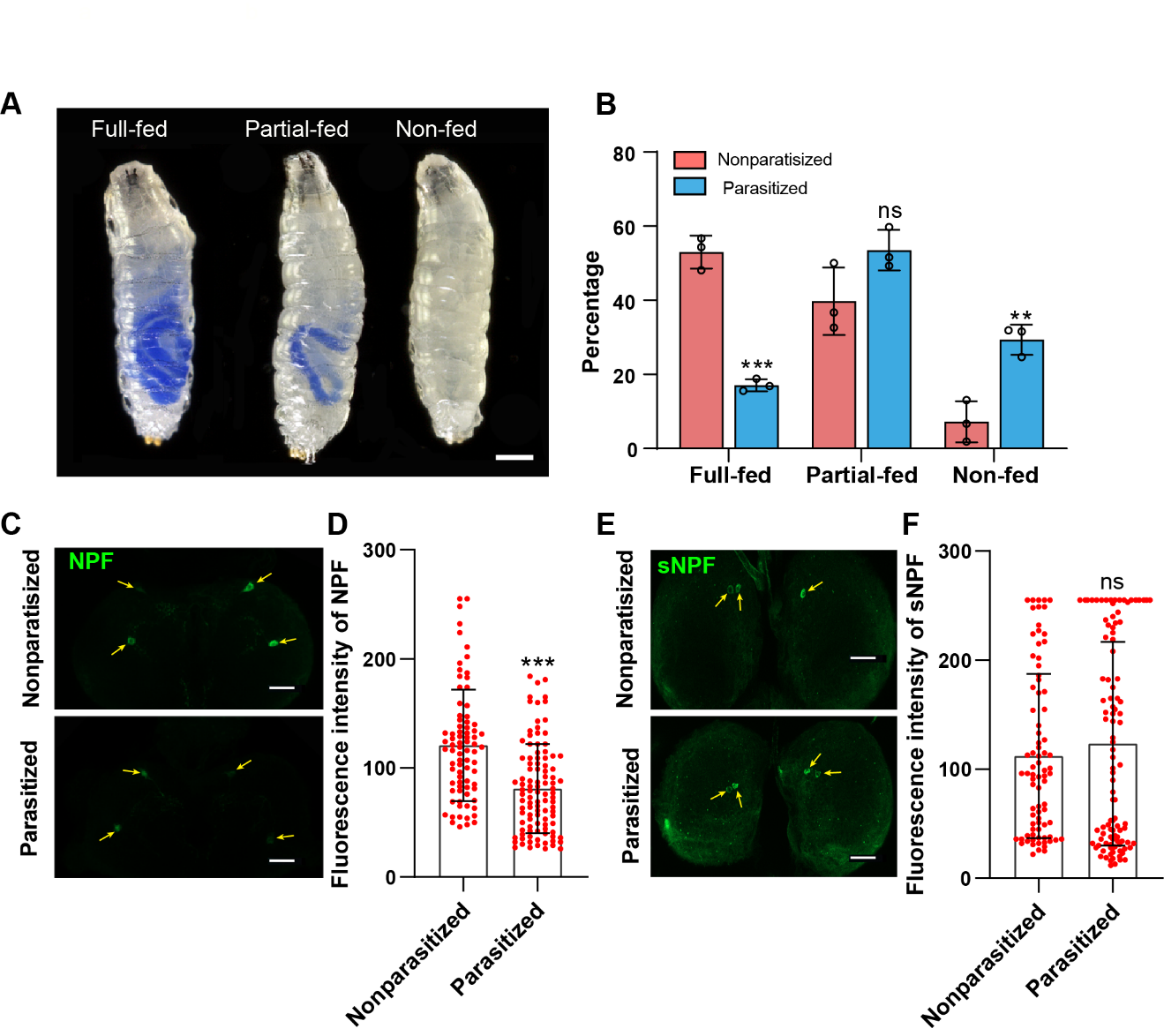
 Suppl. Fig. 6. Parasitism suppresses larval feeding behavior by reducing NPF levels in brain neurosecretory cells.**

**A** After 20 min of feeding on diet containing bromophenol blue, host 3^rd^ instars were categorized as fully-fed, partially-fed or non-fed. Scale bar: 500 μm.

**B** The mean percentage of non-parasitized and parasitized hosts ± SD that were fully, partially or non-fed from three independent replicates of 90 larvae per treatment. Significance was determined by two-sided unpaired Student’s *t* test (ns: non-significant; **: *p* < 0.01; ***: *p* < 0.001).

**C** Immunohistochemical analyses of NPF in brains from non-parasitized and parasitized 3^rd^ instar hosts. Brains were labeled with anti-NPF with stained cells (green) highlighted by yellow arrows. Scale bars: 100 μm.

**D** Fluorescence intensity of NPF in brains from non-parasitized and parasitized hosts. Bars show mean ± SD for cells from 20 larvae per treatment. Significance was determined by two-sided unpaired Student’s *t* test (***: *p* < 0.001).

**E** Immunohistochemical analyses of sNPF in brains from non-parasitized and parasitized host’s 3^rd^ instar hosts. Cells are labeled as described in C. Scale bars: 100 μm.

**F** Fluorescence intensity of sNPF in brains from non-parasitized and parasitized hosts. Bars show mean ± SD for cells from 20 larvae per treatment. Significance was determined by two-sided unpaired Student’s *t* test (ns: non-significant).


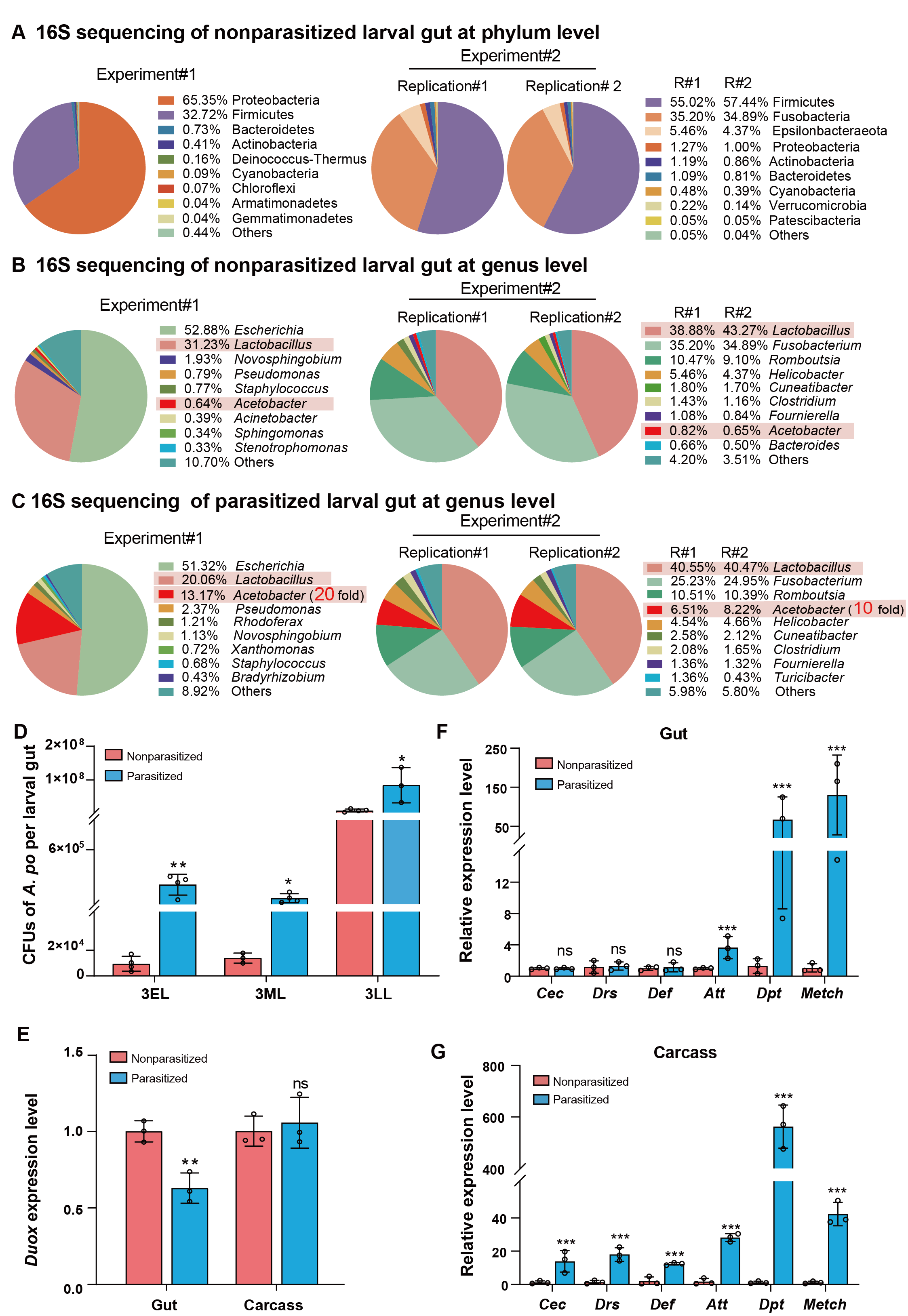
**Suppl. Fig. 7. High throughput sequencing identifies differences in composition of the gut microbiota in unparasitized and parasitized *D. melanogaster* larvae.**

**A, B** The most abundant phyla and genera of bacteria detected in the guts of CN 3^rd^ instar host larvae as determined by 16S rRNA sequencing. The data were generated from early (experiment#1) and late generation (experiment#2) larvae during the study. Data from two replicates were also generated for experiment#2: replication#1 (R#1) and replication#2 (R#2).

**C** Relative gut microbiota abundance at the genus level in parasitized CN host larvae. The data were generated from parasitized host larvae of the same early generation (experiment#1) and late generation (experiment#2) as in (**B**).

**D** CFU counts of *A. pomorum* per gut of early (3EL), middle (3ML) or late (3LL) 3^rd^ instar nonparasitized and parasitized larva (n = 10 for each group). Bars indicate mean ± SD from three independent experiments. Significance was determined by two-sided unpaired Student’s *t* test (*: *p* < 0.05; **: *p* < 0.01).

**E** *Duox* mRNA levels in the guts and carcasses of nonparasitized and parasitized 3^rd^ instar larva (n = 10 for each group). Data were analyzed by Student’s t-test. Values represent mean ± SD of three independent experiments. Significance was determined by two-sided unpaired Student’s *t* test (ns: non-significant; **: *p* < 0.01).

**F, G** RT-qPCR analysis of expression levels of AMPs in the guts (**F**) and carcass (**G**) of nonparasitized and parasitized 3^rd^ instar larva (n = 20 for each group). *Cec*: Cecropin, *Drs*: Drosomycin, *Def*: Defensin, *Att*: Attacin, *Dpt*: Diptericin, *Metch*: Metchnikowin. Data were analyzed by Student’s t-test. Values represent mean ± SD of three independent experiments. Significance was determined by two-sided unpaired Student’s *t* test (ns: non-significant; ***: *p* < 0.001).

**
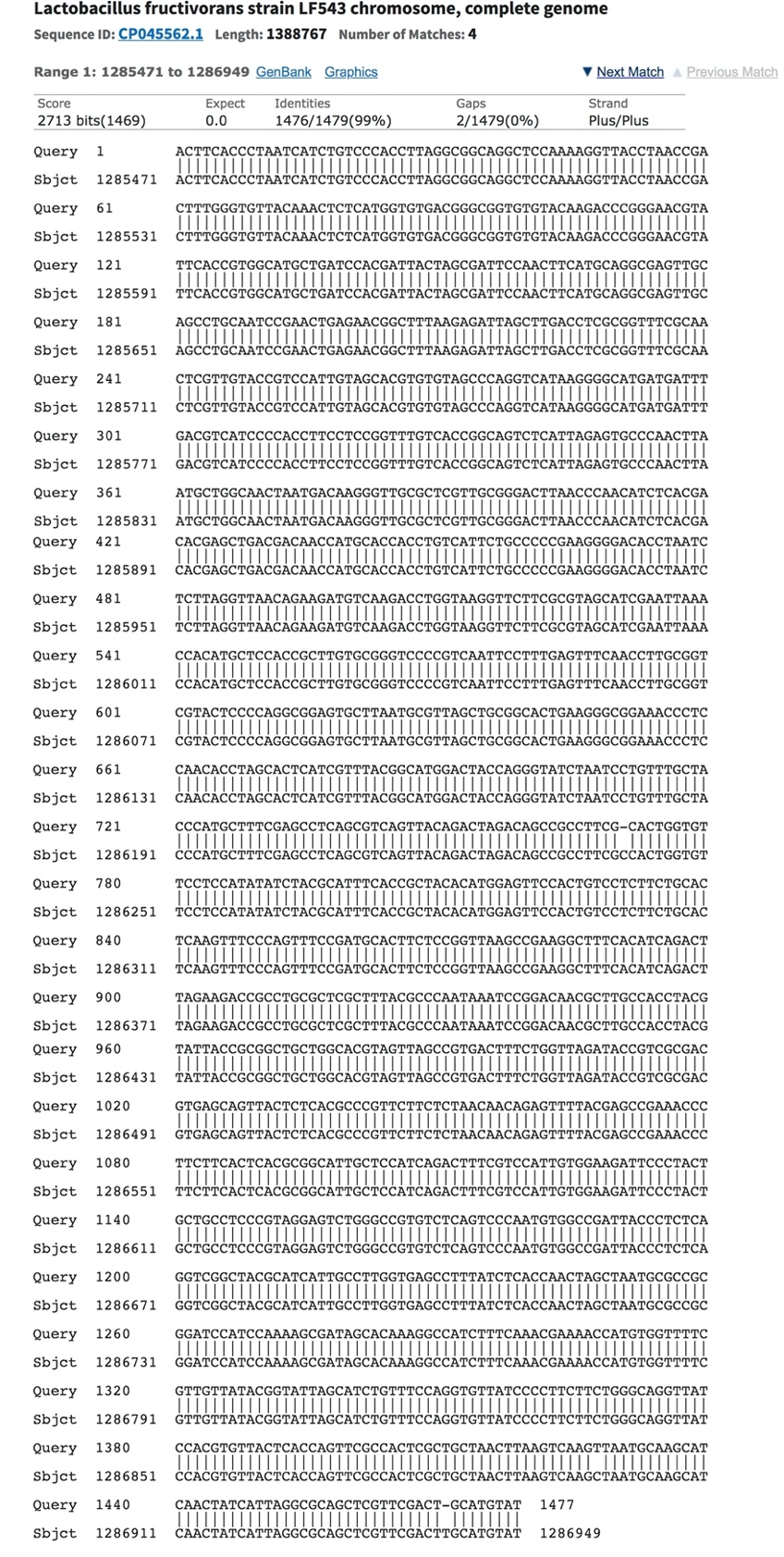
**

**Suppl. Fig. 8. Confirmation of *Lactobacillus fructivorans***. Blast results of sequence data generated by PCR amplification using universal 16S rRNA 27F and 1522R primers. Sequences for the resulting products shared >99% similarity with *Lactobacillus fructivorans***.**

**
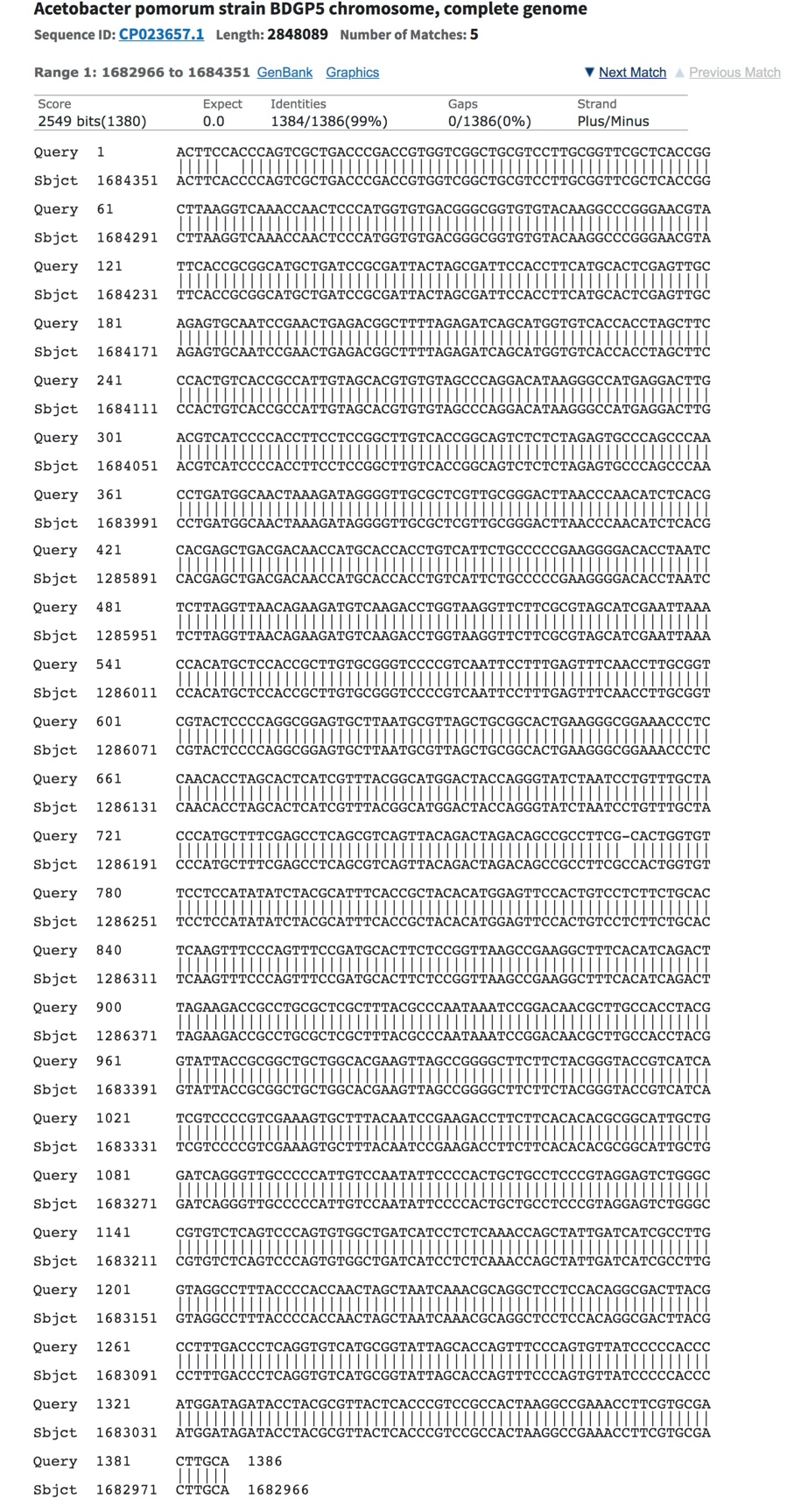
**

**Suppl. Fig. 9. Confirmation** **of *Acetobacter pomorum*.** Blast results of sequence data generated by PCR amplification using universal 16S rRNA 27F and 1522R primers. Sequences for the resulting products shared >99% similarity with *Acetobacter pomorum*.


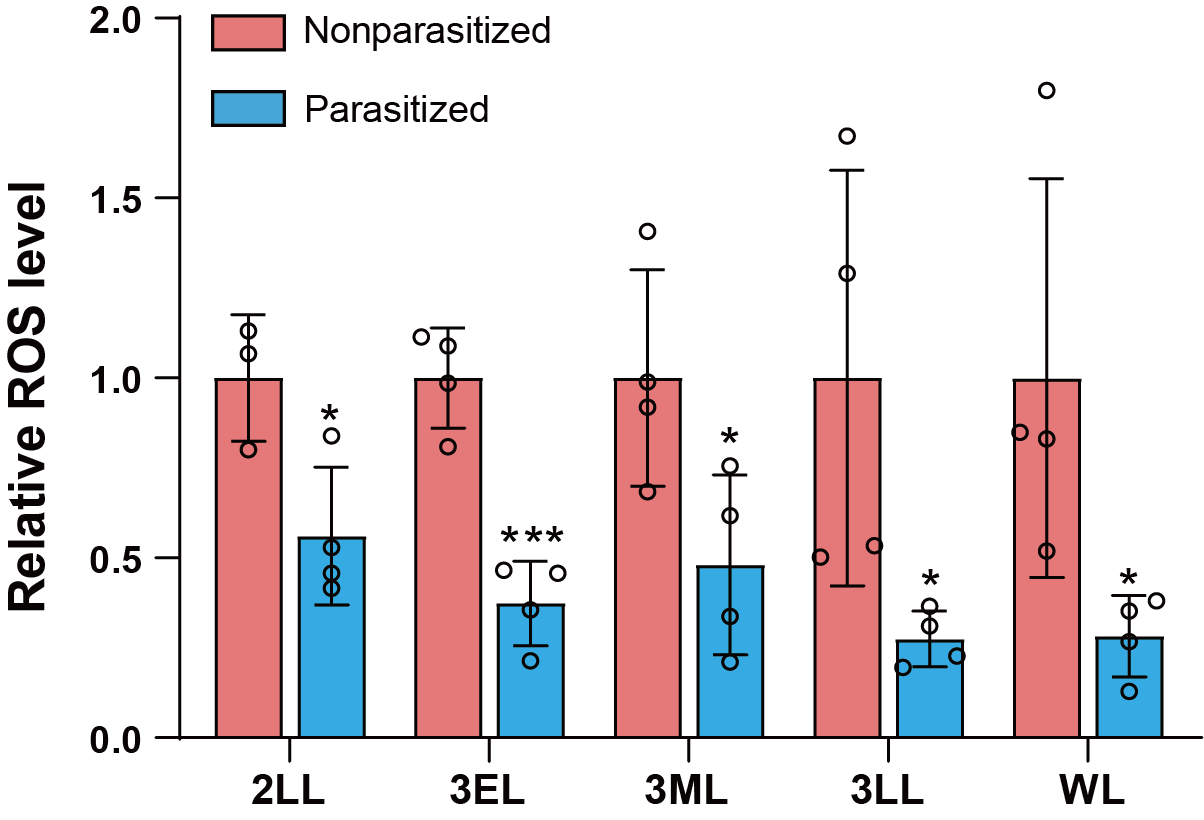


**Suppl. Fig. 10. ROS levels are decreased after parasitism.**

ROS levels in guts of nonparasitized and parasitized hosts within 2LL, 3EL, 3ML, 3LL, and WL different developmental stages, respectively (n=10 for each group). Values represent mean ± SD of three or four independent experiments. Significance was determined by two-sided unpaired Student’s *t* test (*: *p* < 0.05; ***: *p* < 0.001). 2LL: Late 2^nd^ instar Larvae; 3EL: Early 3^rd^ instar Larvae; 3ML: Middle 3^rd^ instar Larvae; 3LL: Late 3^rd^ instar Larvae; WL: Wandering Larvae.


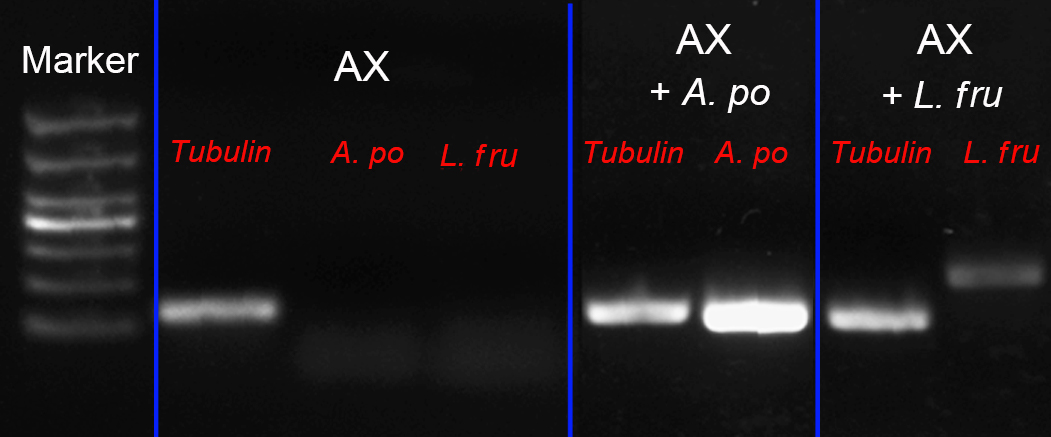


**Suppl. Fig. 11. Generation of gnotobiotic larvae.** PCR analysis of *D. melanogaster* 3^rd^ instar larval gut homogenates using specific primers (n=10 for each group) confirmed that AX larvae contained no bacteria, whereas AX larvae inoculated with *A. pomorum* (AX + *A. po*) or *L. fructivorans* (AX + *L. fru*) produced gnotobiotic larvae. The *D. melanogaster* housekeeping *tubulin* gene was used as the endogenous control.


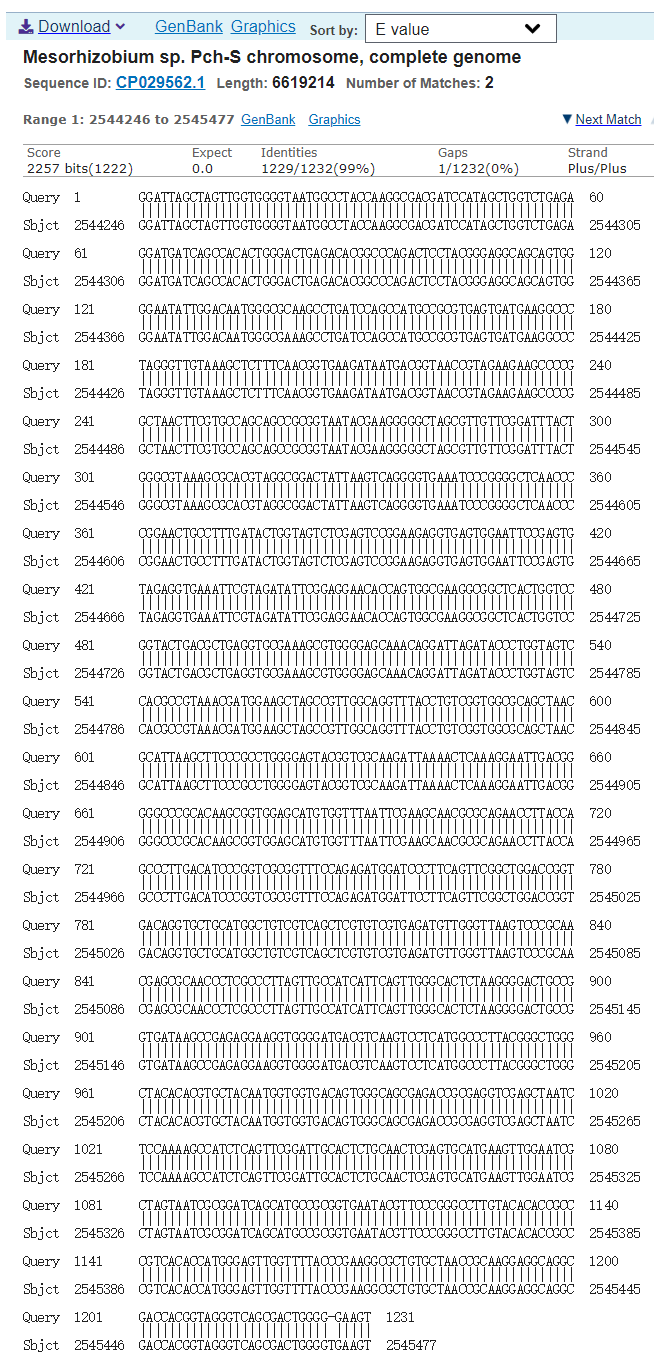


**Suppl. Fig. 12 Confirmation** **of *Mesorhizobium* sp**. Blast results of sequence data generated by PCR amplification using universal 16S rRNA 27F and 1522R primers. Sequences for the resulting products shared >99% similarity with a bacterium in the genus *Mesorhizobium*.


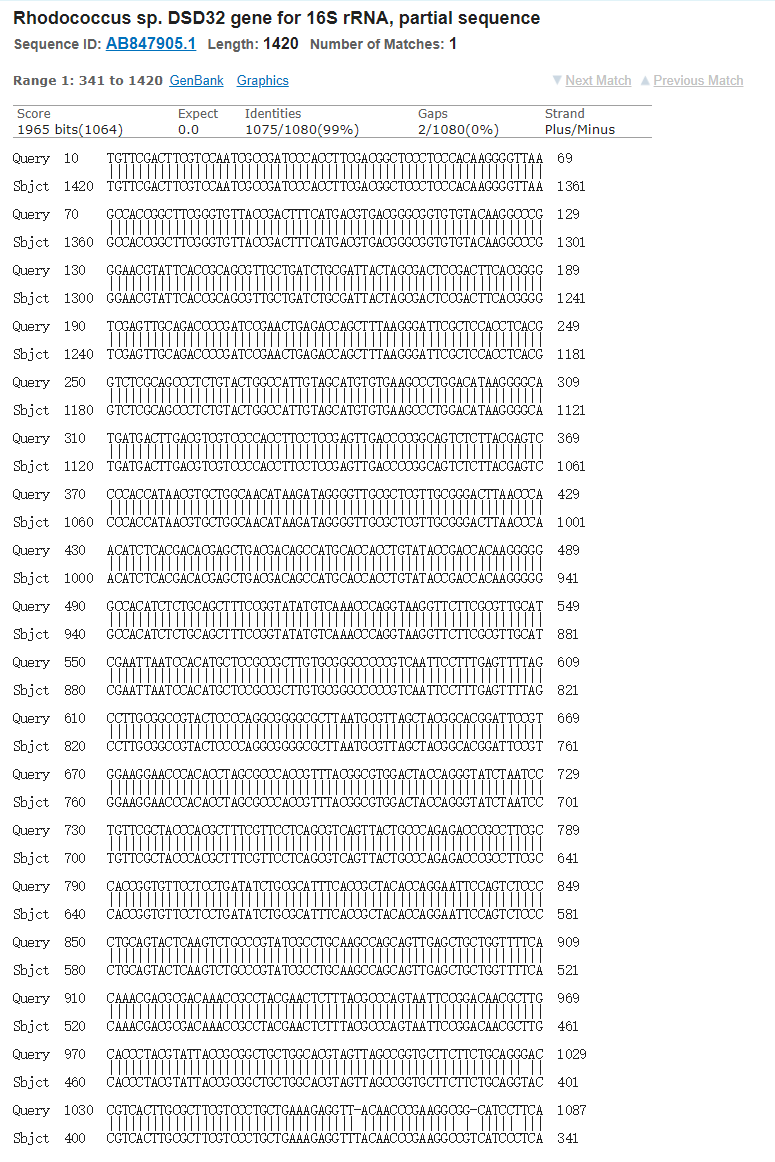


**Suppl. Fig. 13. Confirmation** **of *Rhodococcus* sp**. Blast results of sequence data generated by PCR amplification using universal 16S rRNA 27F and 1522R primers. Sequences for the resulting products shared >99% similarity with a bacterium in the genus *Rhodococcus*.


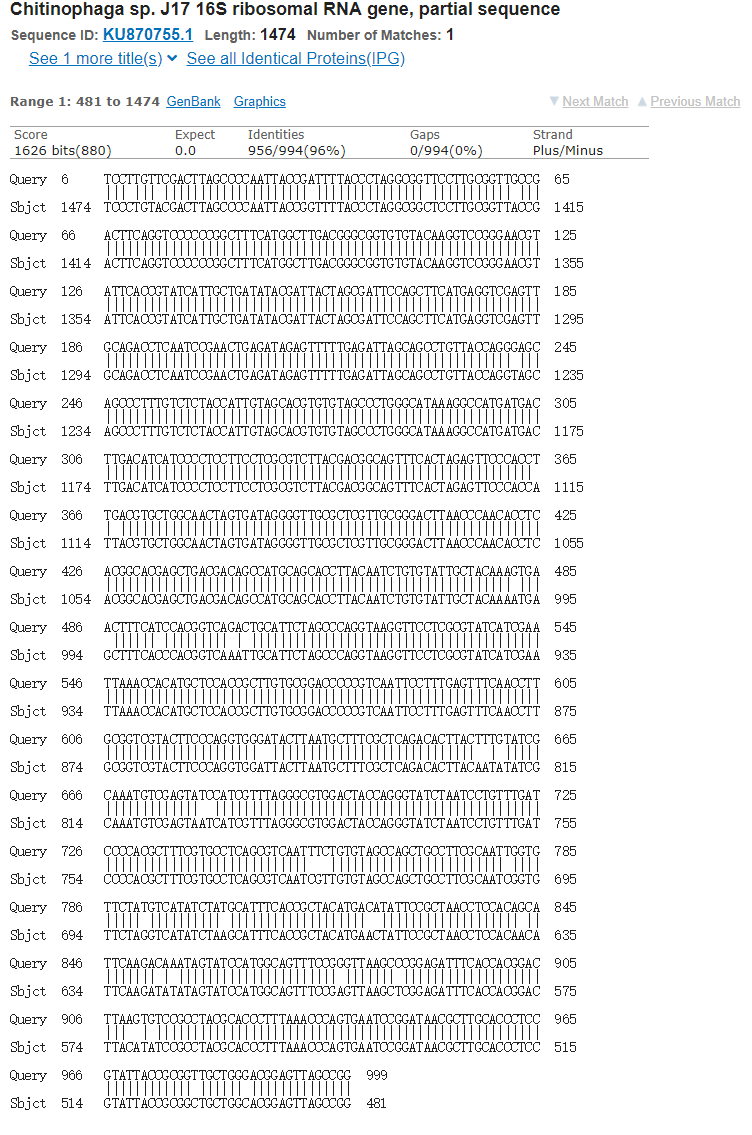


**Suppl. Fig. 14. Confirmation** **of *Chitinophaga* sp**. Blast results of sequence data generated by PCR amplification using universal 16S rRNA 27F and 1522R primers. Sequences for the resulting products shared >96% similarity with a bacterium in the genus *Chitinophaga*.


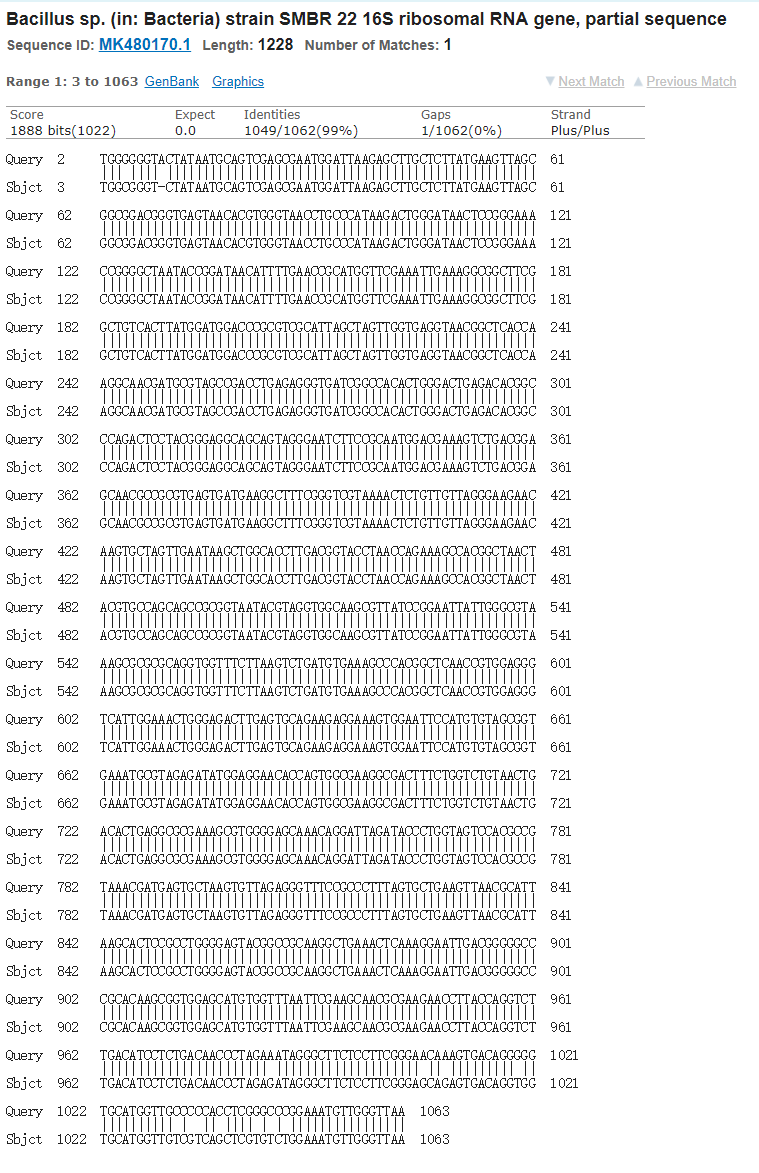


**Suppl. Fig. 15. Confirmation** **of *Bacillus* sp.** Blast results of sequence data generated by PCR amplification using universal 16S rRNA 27F and 1522R primers. Sequences for the resulting products shared >99% similarity with a bacterium in the genus *Bacillus*.


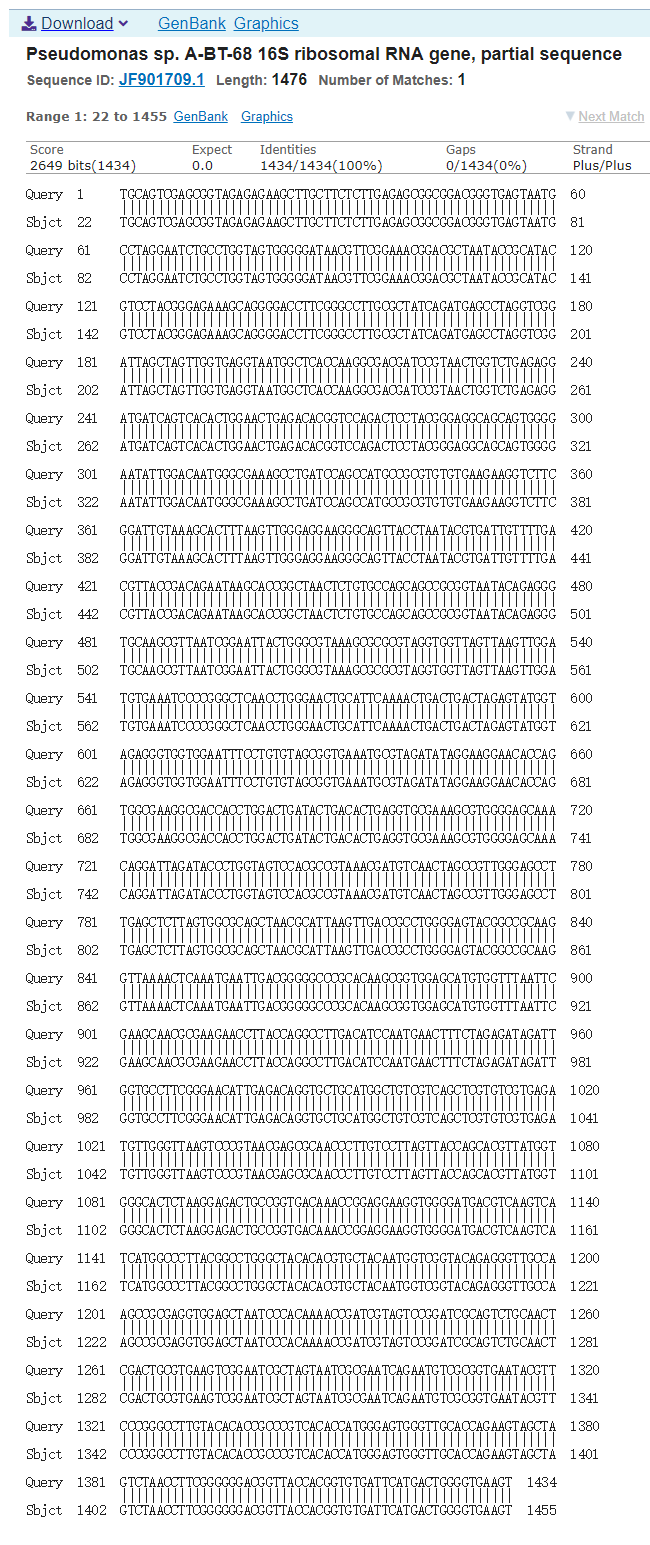


**Suppl. Fig. 16. Confirmation** **of *Pseudomonas* sp.** Blast results of sequence data generated by PCR amplification using universal 16S rRNA 27F and 1522R primers. Sequences for the resulting products shared >99% similarity with a bacterium in the genus *Pseudomonas*.


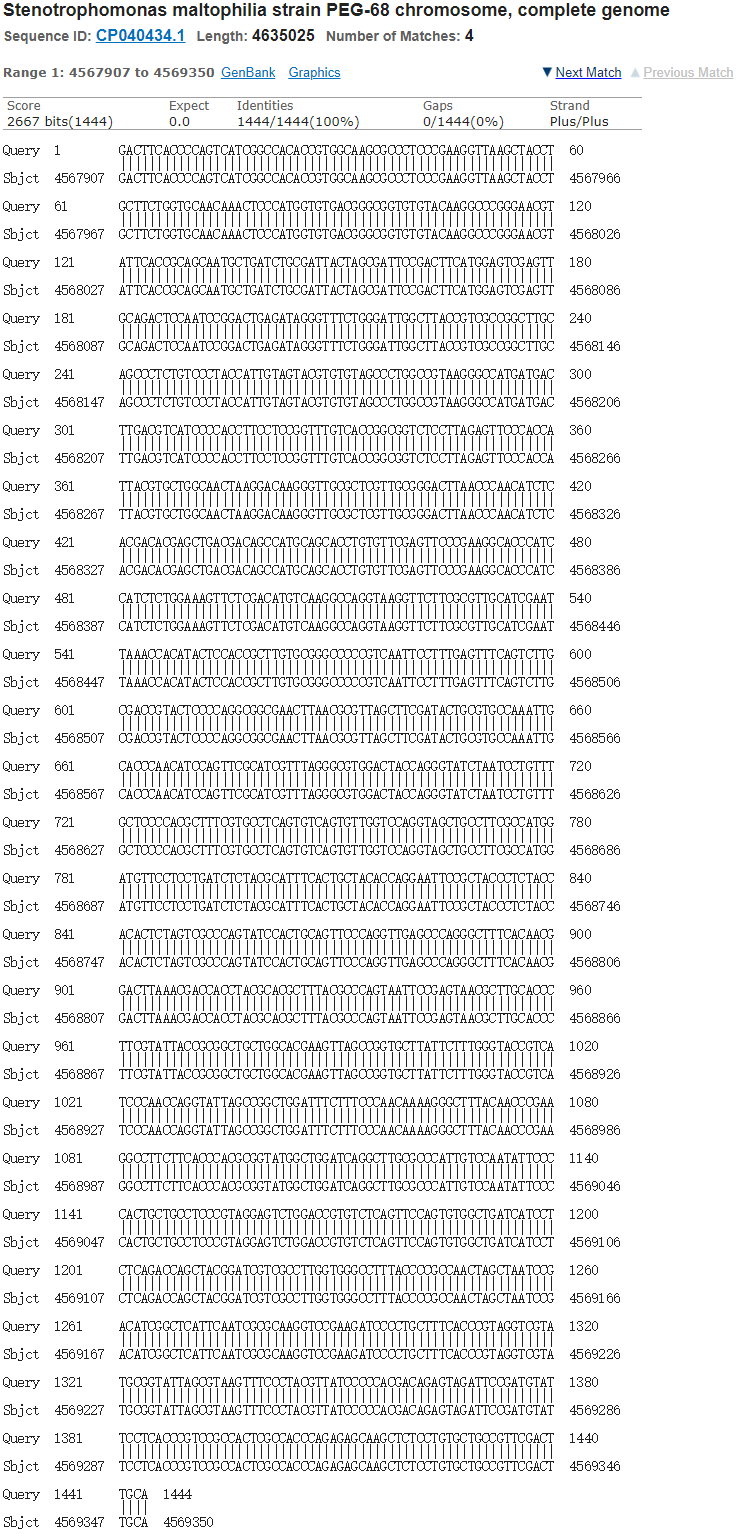


**Suppl. Fig. 17. Confirmation** **of *Stenotrophomonas maltophilia.*** Blast results of sequence data generated by PCR amplification using universal 16S rRNA 27F and 1522R primers. Sequences for the resulting products shared >99% similarity with *Stenotrophomonas maltophilia*.


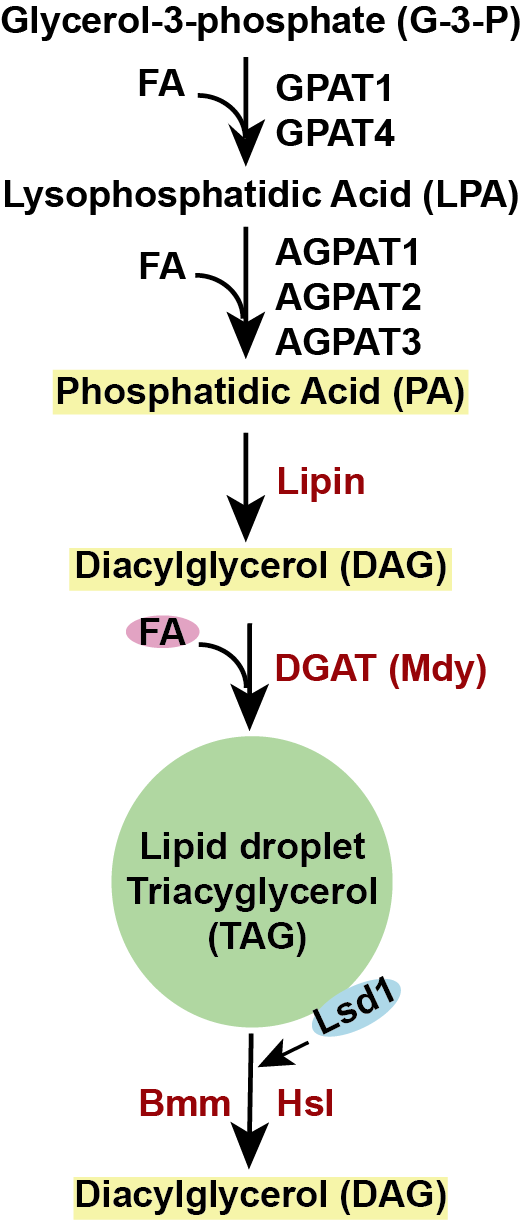


**Suppl. Fig. 18. TAG synthesis and mobilization in *D. melanogaster*.**

Synthesis of LDs occurs through the glycerol-3-phosphate pathway, which converts glycerol-3-phosphate to TAG by esterification with fatty acids in 4 enzymatic steps. In *Drosophila*, Lipin stimulates phosphatidate phosphatase (PAP) activity that converts phosphatidic acid into diacylglycerol (DAG), while DGAT catalyzes the conversion of DAG to TAG in the last step of the lipogenesis pathway. Bmm and Hsl are two key lipases for basal and stimulated lipolysis, respectively. Lsd1 is lipid droplet surface protein that facilitates lipid mobilization.


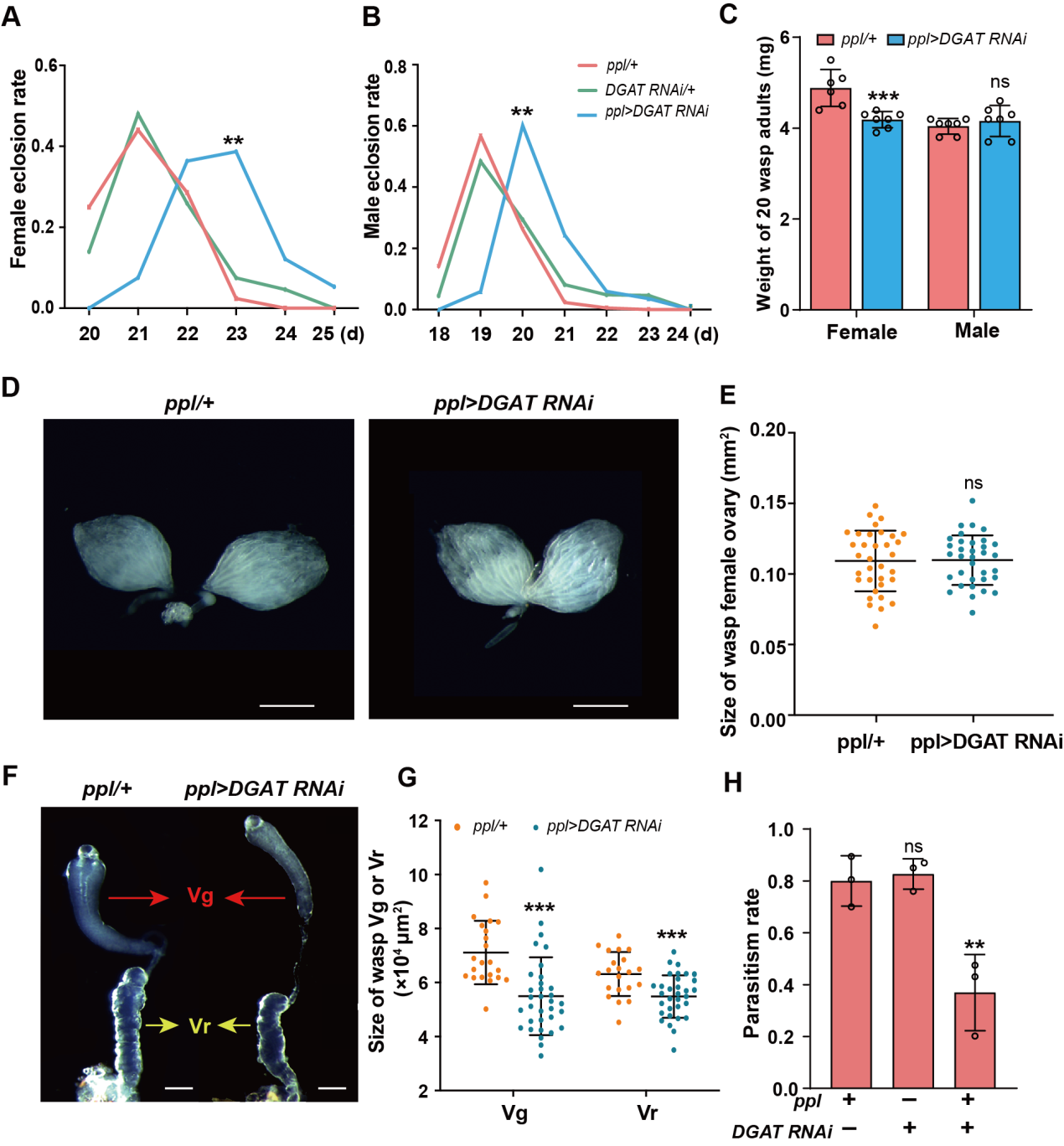


**Suppl. Fig. 19. Extra lipids are critical for development of wasp larvae into adults.**

**A, B** Percentage of Lb female (**A**) and male (**B**) adult wasps that emerge from parasitized *ppl/+*, *DGAT RNAi*/+, and *ppl>DGAT RNAi* hosts (in days). n=200 parasitized hosts for each group. Significance was determined by one-way ANOVA with Tukey’s multiple comparisons test (**: *p* < 0.01).

**C** Pooled weight of 20 female or male wasps that emerged from *ppl/+* and *ppl>DGAT RNAi* hosts. Bars show mean weights ± SD from five independent samples. Significance was determined by two-sided unpaired Student’s *t* test (ns: non-significant; ***: *p* < 0.001).

**D**  Images of paired ovaries from 3-day-old Lb female adults that emerged from *ppl/+* and *ppl>DGAT RNAi* hosts. Scale bars: 200μm.

**E**  Size quantification of ovaries for the treatments shown in D with error bars indicating mean ± SD. n=30 for each group. Significance was determined by two-sided unpaired Student’s *t* test (ns: non-significant).

**F** Venom gland (Vg, red arrows) and venom reservoir (Vr, yellow arrows) from 3-day-old Lb adult female wasps that emerged from *ppl/+* and *ppl>DGAT RNAi* hosts. Scale bars: 100 μm.

**G** Estimated size of the Vg and Vr (n>20 for each sample with error bars showing the mean ± SD). Significance was determined by two-sided unpaired Student’s *t* test (***: *p* < 0.001).

**H** Parasitism rate of female Lb that emerged from *ppl/+*, *DGAT RNAi/+*, and *ppl>DGAT RNAi* hosts (n=200 for each group). Bars show mean proportion of hosts that were parasitized ± SD for each treatment from three independent experiments. Significance was determined by one-way ANOVA with Tukey’s multiple comparisons test (ns: non-significant; **: *p* < 0.01). Genotypes are: *ppl/+* (*ppl-Gal4*/+), *ppl>DGAT RNAi* (*ppl-Gal4*/+; *UAS-DGAT RNAi*/+), *ppl>Bmm RNAi* (*ppl-Gal4*/+; *UAS-Bmm RNAi*/+).

**
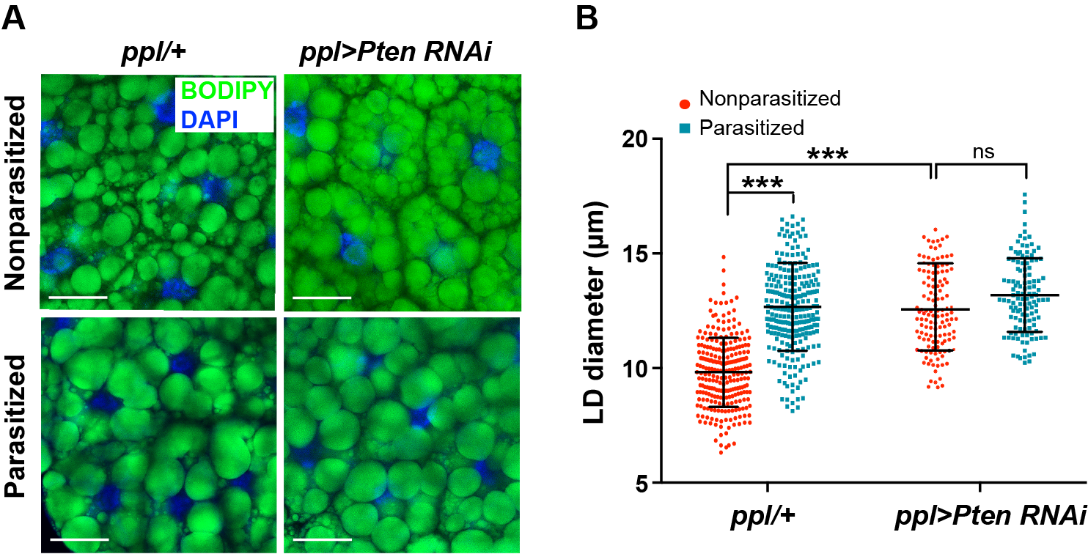
**

**Suppl. Fig. 20. Activation of insulin signaling in parasitized hosts enlarges LDs.**

**A** Fluorescent images of fat bodies from nonparasitized and parasitized hosts. Genotypes are as follows: *ppl/+* (*ppl-Gal4*/+) and *ppl>Pten RNAi* (*ppl-Gal4*/+; *UAS-Pten RNAi*/+). LDs were stained by BODIPY (green) and nuclei were stained by DAPI (blue). Scale bars: 20 μm.

**B** Quantification of LD diameters in (**A**). Each data point is a single LD. A minimum of 100 LDs were measured in fat body cells from 20 individual larvae for each treatment. Error bars show the mean ± SD for each treatment. Significance was determined by two-sided unpaired Student’s *t* test (ns: non-significant; ***: *p* < 0.001).


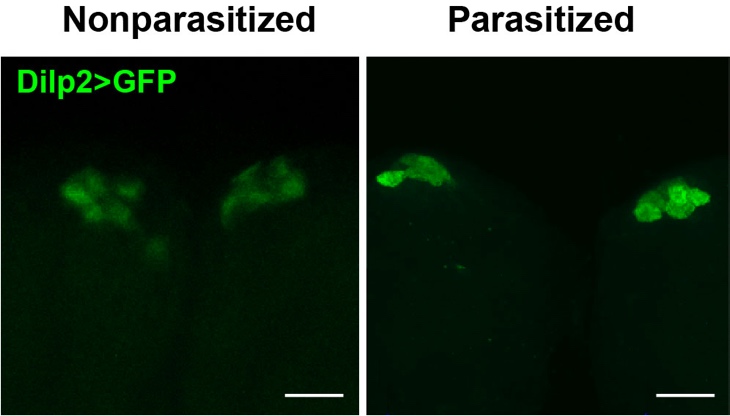


**Suppl. Fig. 21. Parasitism increases Dilp2 expression in IPCs.** Immunohistochemical analysis of Dilp2 (*Dilp2>GFP*, green) in IPCs from non-parasitized and parasitized host’s brains of 3^rd^ instar larvae. *Dilp2>GFP*: *Dilp2-Gal4>UAS-GFP*. Scale bars: 100 μm.

| **Suppl. Table 1. Developmental stages of Lb in relation to developmental stage of *D. melanogaster*.** | | | | | | | | |
| --- | --- | --- | --- | --- | --- | --- | --- | --- |
| Developmental  stage of host | 2^nd^ instar | 3EL | 3ML | 3LL | PP | 1d AP | 2d AP |  |
| Developmental  stage of wasp | Egg | 1^st^ instar | 2^nd^ instar | 3^rd^ instar | | 4^th^ instar | 5^th^ instar |  |

3EL: Early 3rd instar Larvae; 3ML: Middle 3rd instar Larvae; 3LL: Late 3rd instar Larvae; 1d AP: 1 day after host pupation; 2d AP: 2 days after host pupation.

| **Suppl. Table 2. Primers used for 16S rRNA sequencing and PCR assays.** | | | |
| --- | --- | --- | --- |
| **Gene** | **Forward primer (5'-3')** | | **Reverse primer (5'-3')** |
| 515F/926R  (for 16S rRNA sequencing) | GTGCCAGCMGCCGCGGTAA | | CCGTCAATTCMTTTGAGTTT |
| 27F/1522R  (16s primer) | AGAGTTTGATCMTGGCTCAG | | AAGGAGGTGATCCAGCCGCA |
| *A. pomorum*-specific | | CGCAACTGGACACGGACAC | CCCTATACCGACAAGACTGGAGA |
| *L. fructivorans*-specific | | AACAAGAGAAAAAAGAAGCAGGAA | CACTGGCATAAATACAAAAAACAAA |
| *Dilp2* | CGAACTCCTGGACAAACTGC | | CAACGAGGTGCTGAGTATGGT |
| *Dilp3* | AAGCTCTGTGTGTATGGCTT | | AGCACAATATCTCAGCACCT |
| *Dilp5* | GAGTCGCAGTATGCCCTCAA | | AGGACCACTTGGCGGATT |
| *Bmm* | TTTGAGTCACGGGAAGAGGTC | | TAGCGAACGCCACGGAAT |
| *Hsl* | CCTGGAGGCGACCTATGGA | | CGGCGTCTGCTCGTCAAAA |
| *Lsd1* | AGAACCATCGCAGAGGC | | GCGGGTCAGTAGCACAA |
| *Duox* | GCTTACGCCCTGACCTTTG | | ATCCTGCCATCCTGATCCTT |
| *Metch* | GCTGGCAGAGCCTCATCG | | TGGACCCGGTCTTGGTTG |
| *Drs* | CCTTCGCACCAGCACTTCA | | ACTTGTTCGCCCTCTTCGC |
| *Dpt* | CCGTCGCCTTACTTTGCTG | | CCGCCTCCCTGAAGATTGA |
| *Att* | CAATGTGGTGGGTCAGGTTT | | TGTCCGTTGATGTGGGAGTAA |
| *Cec* | GGACAATCGGAAGCTGGGT | | GCAGTTGCGGCGACATT |
| *Def* | CTCAGCCAGTTTCCGATGTG | | AACGCAGACGGCCTTGTC |
| *AkhR*  (RNAi validation) | CAGGAGCGACTTTGATGAGAA | | TGGCTCCGTAGCAGTAGATGA |
| *InR*  (RNAi validation) | TCGCATCGGAGTGACTGGTA | | ATGTCACGGGTCATTCCAAAG |
| *DGAT*  (RNAi validation) | GCCTTAGCCACTGAGCGACTT | | CCATTTGGACCACAGGGATGT |
| *Lipin*  (RNAi validation) | AGTGCTCAGGAGTCGGGAGA | | CCAAGAAGCTGTTGGGTATGG |
| *Pten*  (RNAi validation) | CTTCTCGGAGTCCAGTTGTGTG | | GGTTTTCAGTCTATCTGGCTTTGC |
| *Bmm*  (RNAi validation) | AGTTTGAGTCACGGGAAGAGGTC | | TGTAGCGAACGCCACGGAAT |
| *Hsl*  (RNAi validation) | ACCCGTTCCACCATATTCTTCC | | GCGTATCGAGTCGCCATACTGA |
| *Lsd1*  (RNAi validation) | GGCGTTCTATGGTAGCCTTCA*G* | | ATCGCTGCCCGTCACCTTCT |
| *Tubulin* | GTGGACTCAGTGCTCGATGT | | AATCAGCAGGGTTCCCATAC |
